# Application of *in vivo* solid phase microextraction (SPME) in capturing metabolome of apple (*Malus* ×*domestica* Borkh.) fruit

**DOI:** 10.1038/s41598-020-63817-8

**Published:** 2020-04-21

**Authors:** Sanja Risticevic, Erica A. Souza-Silva, Emanuela Gionfriddo, Jennifer R. DeEll, Jack Cochran, W. Scott Hopkins, Janusz Pawliszyn

**Affiliations:** 1 Department of Chemistry, University of Waterloo, 200 University Avenue West, N2L 3G1 Waterloo, Ontario Canada; 2 Departamento de Química, Universidade Federal de São Paulo (UNIFESP), Rua São Nicolau, 210, 09913-030 Diadema, São Paulo Brazil; 3 Department of Chemistry and Biochemistry, University of Toledo, 2801 W. Bancroft St., 43606-3390 Toledo, Ohio USA; 4 Ontario Ministry of Agriculture, Food and Rural Affairs, 1283 Blueline Rd. at Hwy #3, Box 587, N3Y 4N5 Simcoe, Ontario Canada; 5 VUV Analytics, 715 Discovery Blvd, Ste 502, 78613 Cedar Park, Texas USA

**Keywords:** Analytical chemistry, Mass spectrometry

## Abstract

An *in vivo* direct-immersion SPME sampling coupled to comprehensive two-dimensional gas chromatography – time-of-flight mass spectrometry (GCxGC-ToFMS) was employed to capture real-time changes in the metabolome of ‘Honeycrisp’ apples during ripening on the tree. This novel sampling approach was successful in acquiring a broad metabolic fingerprint, capturing unique metabolites and detecting changes in metabolic profiles associated with fruit maturation. Several metabolites and chemical classes, including volatile esters, phenylpropanoid metabolites, 1-octen-3-ol, hexanal, and (2*E*,4*E*)-2,4-hexadienal were found to be up-regulated in response to fruit maturation. For the first time, Amaryllidaceae alkaloids, metabolites with important biological activities, including anti-cancer, anti-viral, anti-parasitic, and acetylcholinesterase (AChE) inhibitory activity, were detected in apples. Considering the elimination of oxidative degradation mechanisms that adversely impact the representativeness of metabolome obtained *ex vivo*, and further evidence that lipoxygenase (LOX) pathway contributes to volatile production in intact fruit, *in vivo* DI-SPME represents an attractive approach for global plant metabolite studies.

## Introduction

In food analysis, there is increasing interest in the implementation of metabolomics approaches to understand complex biological networks that control production of high-quality food commodities and crop plants, from both health- and safety-related aspects. In metabolomics applications, great care should be expended when selecting sample preparation and extraction methods for a given application, as the employed methods play a large role in determining metabolome coverage, and subsequently, the quality of the attained data^1^. In this regard, attainment of comprehensive metabolite coverage necessitates implementation of non-selective and unbiased sample preparation and extraction methods. Further, as an essential component of such methods, metabolomics sampling and sample preparation protocols must incorporate a suitable metabolism-quenching step to terminate enzymatic activity, prevent enzyme-mediated metabolite conversions, and eliminate chemical breakdown of labile metabolites. Metabolism quenching of plant tissues is traditionally carried out by methods such as freezing with liquid nitrogen, freeze-drying, and addition of alcohol or acid^2^. However, implementation of these traditional methods may result in alterations to the metabolomic profile due a multiplicity of factors, such as metabolite decompositions and interconversions, losses of volatile metabolites, emission of touch- or wound-induced metabolites, and non-reversible losses of metabolites by absorption to cell walls and membranes^3–6^. Hence, it becomes questionable whether the metabolome captured under such circumstances is a true signature of the biochemical activity of the investigated system.

Given the importance of apples as a valuable food crop with high worldwide demand, several studies have taken as focus the profiling of volatile metabolites in apples with respect to a multitude of factors that determine their nutritional quality, market desirability, and safety for human consumption. Notable examples include studies targeted at examination of the genetic and molecular basis of apple aroma, characterization of apple varieties according to skin colour and origin, investigation of postharvest quality of integrated and organically produced apple fruit, elucidation of volatile profile with respect to fruit ripening, and the development of disorders (such as superficial scald and bitter pit)^7–12^.

In solid phase microextraction (SPME), analytes are extracted by an extraction phase that is exposed directly to the sample matrix or to the headspace above it^13–15^. In recent years, SPME has been increasingly employed for *in vivo* analysis of biological systems owing to desirable SPME properties such as solvent-free sample preparation, miniaturized format, and non-exhaustive analyte recovery^15^. Theoretically, the amount of analyte extracted by SPME becomes independent of sample volume when large sample volumes are analyzed, or in cases where compounds with low fiber coating/sample matrix distribution constants (*K*_*fs*_) are targeted^14^; accordingly, under these conditions, negligible analyte depletion can be assumed to transpire in the studied sample. Owing to the small dimensions of the SPME sampling device and the negligible non-exhaustive analyte recovery feature, metabolome perturbations incurred during the sampling of living systems are thus minimal^14^. In this respect, *in vivo* SPME has been effectively utilized for determination of environmentally relevant compounds and global metabolomic fingerprinting in tissues and biological fluids of living, freely moving animals, as well as in determinations of biologically active compounds in plant, insect, and animal emissions^15–22^.

In this study, *in vivo* DI-SPME has been employed for high-resolution untargeted metabolomics profiling of ‘Honeycrisp’ apples (*Malus* ×*domestica* Borkh.). The sample preparation protocol was designed to provide a real-time snapshot of endogenous volatile and semivolatile metabolome, since endogenous metabolite composition is directly related to gene expression^23^. As opposed to our previously published study, which had as focus a global evaluation of analytical performance and precision of *ex vivo* and *in vivo* SPME platforms for determination of volatile and semivolatile apple metabolites, the current study aims to determine the suitability of *in vivo* DI-SPME for obtaining broad and representative metabolome coverage, and for detection of changes in metabolic profiles of apples characterized by different maturities.

## Results

### *In vivo* DI-SPME as a tool to characterize the metabolome of apples from different maturity stages

Biosynthesis of volatile metabolites is one of the key contributors to the final sensory quality of fruit produce^24,25^. The blend of volatiles produced by fruit is species- and cultivar-specific, as well as representative of its development stages, undergoing distinguishable changes throughout fruit growth and ripening^25,26^. Given that gene expression and associated enzyme and metabolite complements change during fruit development and maturation, the maturity of the fruit at the time of harvest is the major factor influencing volatile metabolome composition^27^. Further, although the majority of published studies related to this topic have been concerned with the ripening of harvested apples, the rate of ripening has been shown to be slower for apples still on the tree as compared to harvested apples^28^.

In the current work, the levels of 225 volatile and semivolatile metabolites determined by *in vivo* DI-SPME (sampling season 2010) were used to characterize samples according to maturity stage through application of multivariate statistical analysis tools such as principal component analysis (PCA) and orthogonal partial least squares discriminant analysis (OPLS-DA). Supplementary Fig. 1 shows the PCA scores plot of the two major principal components, which explain 70.8% of the overall variance. As can be seen, clear differentiation is observed between metabolite profiles obtained via *ex vivo* HS-SPME and those obtained via *in vivo* SPME. While SPME sampling mode was the main factor for sample differentiation along PC2, with distinct metabolite profiles obtained with *ex vivo* and *in vivo* SPME, discrimination along PC1 was given mainly by fruit maturity.

**Figure 1:**
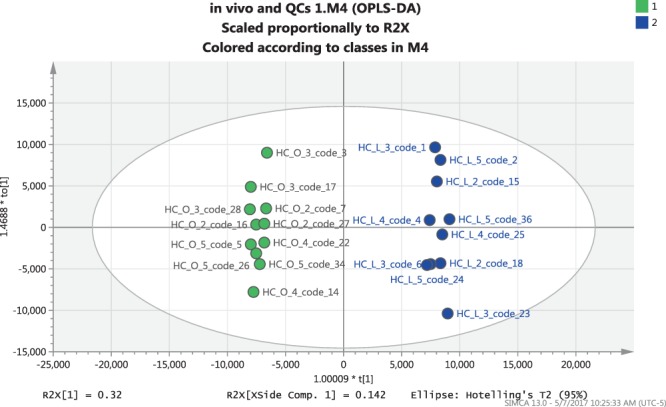
Scores plot corresponding to OPLS-DA analysis of *in vivo* DI-SPME data for HC-L apples (higher maturity index, represented by blue circles) and HC-O apples (lower maturity index, represented by green circles).

**Figure 2:**
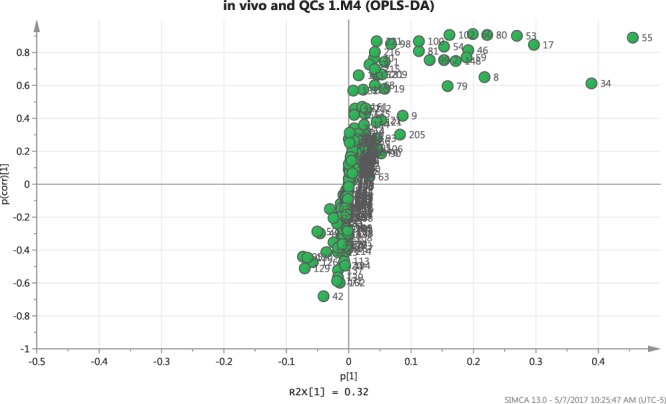
S-plot corresponding to *in vivo* DI-SPME data for 225 metabolites in HC-L apples (higher maturity index) and HC-O apples (lower maturity index).

In order to acquire a higher level of group separation and enable identification of variables responsible for differentiation, OPLS-DA, a supervised technique, was applied to the attained data. As can be seen from the OPLS-DA score plot presented in Fig. 1, significant variations in the contents of volatile and semivolatile metabolites were induced by maturity level. The interpretation of the loading plot, more specifically the S-plot (Fig. 2), enabled identification of the most statistically significant variables for differentiation of samples according to maturity level. The variables with highest weight in the groups separation presented by the model are butyl 2-methylbutanoate, ethyl hexanoate, propyl 2-methylbutanoate, ethyl 2-methylbutanoate, ethyl butanoate, pentyl 2-methylbutanoate, ethyl propanoate, butyl 2-methylpropanoate, 1-propylethanoate, butyl butanoate, 1-methoxy-4-(2-propenyl)benzene (estragole), hexyl 2-methylbutanoate, propyl butanoate, butyl propanoate, (*E*)-1-methoxy-4-(1-propenyl)benzene (*trans*-anethole), propyl propanoate, 1-methoxy-4-(1*Z*)-1-propenyl-benzene (*cis*-anethole), 5-hexenyl acetate, 1-octen-3-ol, 2-methylbutyl acetate, butyl acetate, hexanal, (2*E*,4*E*)-2,4-hexadienal (sorbic aldehyde), and two unknowns (Supplementary Table 1).

### *In vivo* SPME sampling of apples: metabolome coverage and comparison to *ex vivo* DI-SPME sampling

The contour plots of the GCxGC-ToFMS total ion current (TIC) chromatograms corresponding to two different SPME sampling approaches employed during the 2011 sampling season are illustrated in Supplementary Fig. 2. Substantially differing GCxGC-ToFMS profiles were obtained depending on the mode of SPME sampling that was employed. Over 300 volatile and semivolatile compounds, including alcohols, aldehydes, ketones, esters, acids, ethers, hydrocarbons, and terpenes have been previously reported as components of the apple metabolome^25,27,29^. The results herein obtained for *in vivo* DI-SPME sampling of intact apple fruit corroborate previous reports, showing that esters are the most important contributors to the aroma profile of apples, in both quantitative and qualitative terms^27^. Based on a visualization of structurally ordered GCxGC chromatograms, the ester complement in the metabolome profile of the ‘Honeycrisp’ apple variety includes 13 acetates, 5 propanoates, 10 butanoates, and ethyl hexanoates (Supplementary Table 2). In addition, the comprehensive metabolome coverage obtained with *in vivo* SPME is reflected by the extraction of aldehydes, ketones, alcohols, aromatic compounds, carboxylic acids, aromatic aldehydes, aromatic ketones, benzyl alcohols, alkanes, benzyl acetates and glycol ethers, as well as metabolites originating from the lipoxygenase (LOX) pathway (Supplementary Table 2). A peak apex plot illustrating elution time coordinates of tentatively identified metabolites grouped in respective homologous compound series is presented in Supplementary Fig. 3. A previous study by our group indicated that the manifestation of analyte peaks exhibiting overloaded and streaking profiles was related to the formation of GC-amenable products of Maillard reaction during thermal desorption^30–34^. Their production was significantly reduced when PDMS-overcoated DVB/CAR/PDMS fiber coatings were used for *in vivo* extraction due to the antifouling properties of these SPME coatings^33^. Moreover, whenever present, these artefact compounds were excluded from the data set prior to statistical analysis.

In addition to its concentration, the contribution of each compound to the characteristic aroma profile of a given fruit is also modulated by other factors, such as the overall fruit composition, as well as the odor threshold above which the compound can be detected by smell^24–26^. Based on the odor thresholds cited in the literature, character impact odorants for apple fruit are ethyl butanoate, ethyl 2-methylbutanoate, 2-methylbutyl acetate, ethyl hexanoate, hexyl acetate, hexyl propanoate, and hexyl 2-methylbutanaote^27,35^. Among these, low levels of ethyl butanoate and ethyl hexanoate were found in the volatile fraction of ‘Golden Reinders’ apples at harvest^35^. For ‘Mondial Gala’ apples, low levels of ethyl butanoate and ethyl 2-methylbutanaote were detected at harvest^27^. Although absolute quantification of compounds was outside of the scope of the current *in vivo* DI-SPME metabolomics study, the results herein obtained demonstrate that the proposed analytical approach has sufficient sensitivity to capture specific character impact odorants, including those that are commonly present at low concentrations in several investigated apple cultivars in literature, including ‘Golden Reinders’ and ‘Mondial Gala’ apples (Supplementary Table 2).

**Figure 3:**
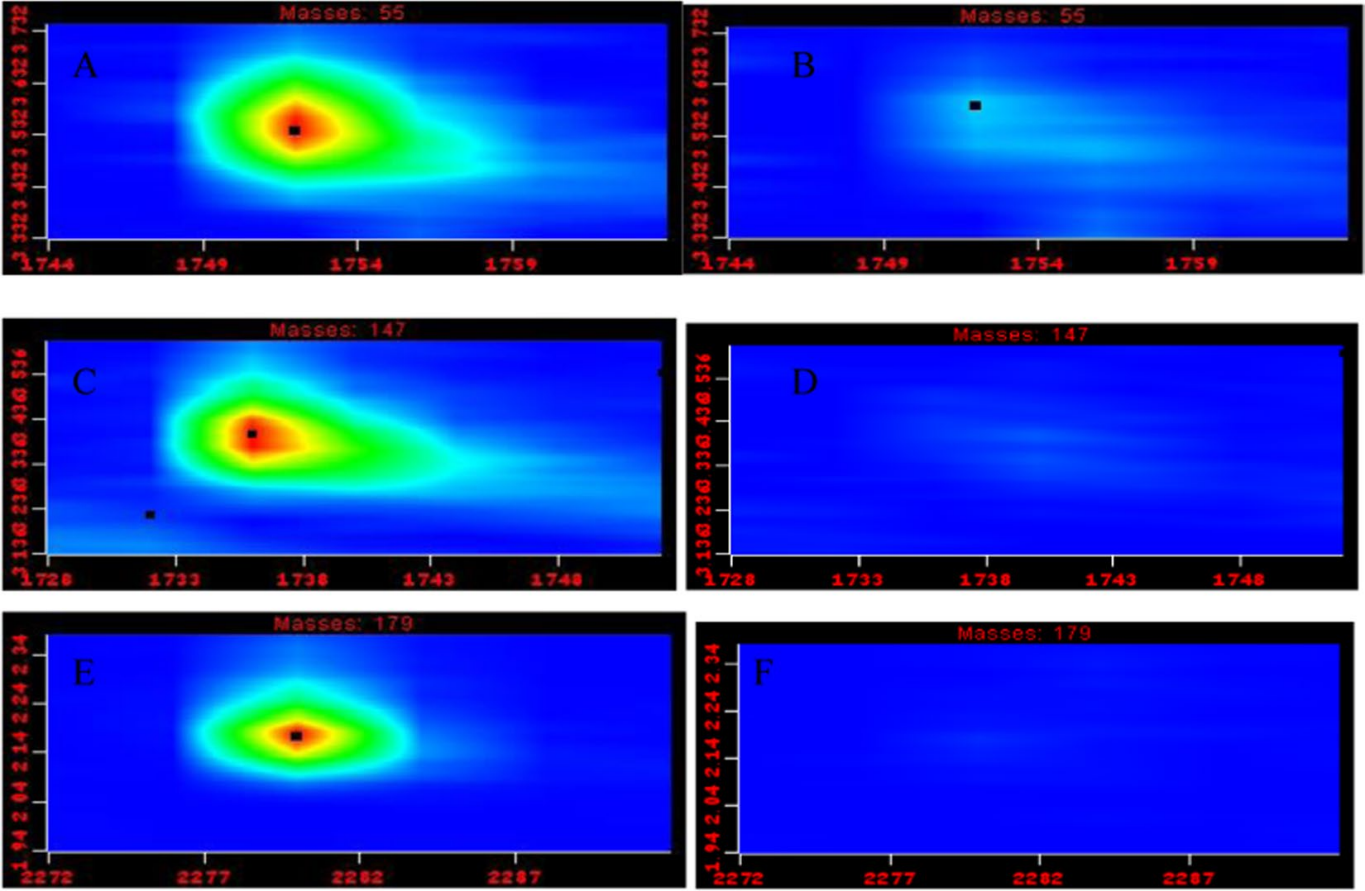
Contour plots of GCxGC extracted ion chromatograms corresponding to elution windows of metabolites unique to *in vivo* approach in *in vivo* (left plots) and *ex vivo* (right plots) extracts. (**A**,**B**) unidentified analyte 1 (hit #1:1-Hepten-4-ol), (**C**,**D**) 1,4-Diacetylbenzene (p-Acetylacetophenone), (**E**,**F**) unidentified analyte 2 (hit #1:2-(4-tert-Pentylphenoxy)ethanol).

In order to draw a comparison of metabolite coverage between *in vivo* and *ex vivo* SPME, each individual metabolite present in final data compilations was manually aligned in all samples involved in the comparative study. In total, three metabolites (Supplementary Table 3) were unique to the profiles attained via the *in vivo* SPME mode of extraction. Their GCxGC contour profiles and mass spectra are presented in Fig. 3 and Supplementary Fig. 4, respectively. On the other hand, the number of specific metabolites that were unique to metabolite profiles attained via *ex vivo* DI-SPME was significantly higher. This group of unique compounds includes (2*E*)-2-heptenal, (2*Z*)-2-octenal, (2*E*,4*E*)-2,4-nonadienal, (2*E*,4*E*)-2,4-heptadienal, (3*E*,5*E*)-3,5-octadien-2-one and (2*E*,6*Z*)-2,6-nonadienal. (Supplementary Fig. 5). Metabolomic profiles corresponding to *in vivo* and *ex vivo* SPME sampling during two consecutive sampling seasons (2010 and 2011) confirmed that these metabolites were either not detected or only present at trace levels when the *in vivo* sampling mode was used.

## Discussion

Straight chain esters, branched chain esters, estragole, *trans*-anethole, *cis*-anethole, 1-octen-3-ol, hexanal and (2*E*,4*E*)-2,4-hexadienal were found to be upregulated in more mature apples, as shown by the box plots presented in Supplementary Fig. 6. Corroborating the associated literature, fruits with a lower maturity index showed lower capacity for volatile biosynthesis due to the absence of volatile precursors and enzyme-forming systems, since the ester-forming enzyme system is induced only during the later stages of fruit maturation^24,25,28^.

**Figure 4:**
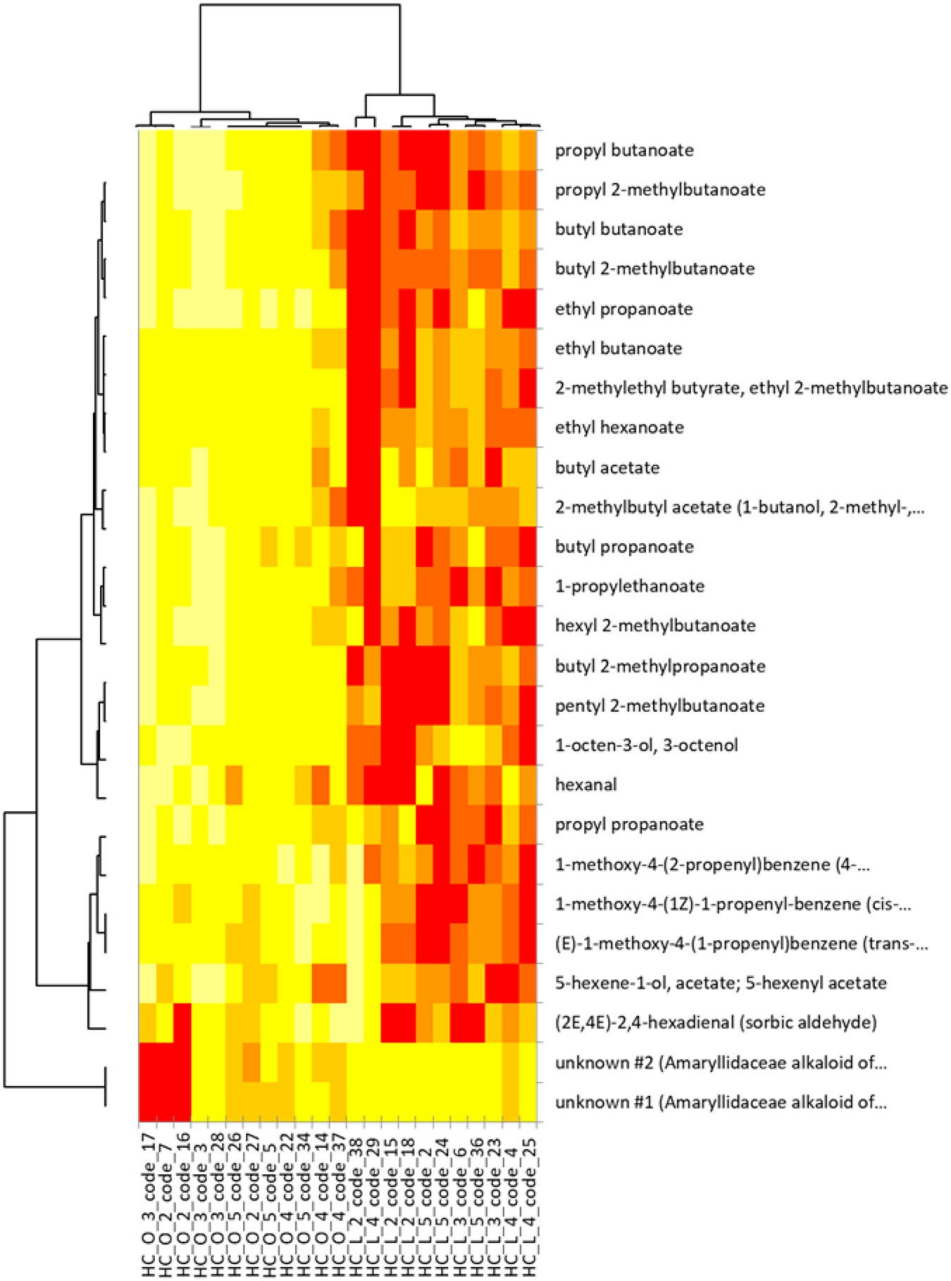
Heat map of metabolite abundances (*y*-axis) obtained by *in vivo* DI-SPME for ‘Honeycrisp’ apples (*x*-axis) of higher harvest maturity (HC-L) and lower harvest maturity (HC-O). Metabolites expressed in higher quantities are expressed in red colour, while metabolites expressed in lower quantities are expressed in light yellow colour.

Aiming to further explicate the apple metabolomics data, in addition to PCA and OPLS-DA, a heat map with hierarchical clustering analysis was used as a complementary multivariate analysis approach (Fig. 4). These results indicate that ripening results in an enhancement of volatile ester-synthesizing capacity and up-regulation of a specific blend of metabolites characteristic to the ‘Honeycrisp’ cultivar. These specific metabolites may not be regulated to the same extent by ripening-related metabolic processes in other apple cultivars, since substantial differences in metabolite profiles produced during ripening were detected even among cultivars having significant phenotypic similarities^25^. For example, differences in degree of ethyl ester enhancement among cultivars were reported to be the result of differential synthesis or activity of alcohol acyl CoA transferase (AAT), or alcohol dehydrogenase (ADH), variation in contents of alcohol precursors, separate iso-forms of AAT and ADH having different substrate specificities^36^. Considering these cultivar-related differences and that volatile and semivolatile metabolome composition plays a crucial role in definition of fruit ripeness, maturity index, and characterization of physiological stage of fruit development, the ripening-dependent up-regulation of metabolites should be considered more fundamentally.

For apple, a blend of volatile compounds composed of alcohols, aldehydes, ketones, sesquiterpenes, esters, and polypropanoids is produced from primary metabolites via at least four pathways. Straight chain esters such as ethyl hexanoate, ethyl butanoate, ethyl propanoate, 1-propylethanoate, butyl butanoate, propyl butanoate, butyl propanoate, propyl propanoate, butyl acetate (Supplementary Table 1) are synthesized by lipids via β-oxidation and lipoxygenase/hydroperoxide lyase (LOX/HPL) pathways^12,37^. Metabolism of fatty acids through β-oxidation, hydroxyacid cleavage, and lipoxygenase/hydroperoxide lyase pathways intensifies during fruit maturation, playing a key role in biosynthesis of precursors required for straight-chain ester formation^24–26,35,36^. At early maturity stages, enzymes and substrates of the LOX pathway are located in different subcellular sites; accordingly, lipid metabolism increases with respect to ripening as cell walls and membranes become more permeable to different substrates^24,25^. Enhancements in biosynthesis of volatile esters, including butyl 2-methylbutanoate, hexyl 2-methylbutanote, hexyl hexanoate, hexyl butanoate, ethyl hexanoate, hexyl acetate, and 2-methylbutyl acetate, were detected for ‘Golden Reinders’, ‘Pink Lady’, and ‘Fuji’ apple fruit^24–26^.

On the other hand, the HPL biosynthetic pathway is responsible for cleavage of fatty acid hydroperoxides originated through LOX action into aldehydes, alcohols, and volatile esters^24–26^. HPL activity in flesh tissue was found to be correlated to regeneration of fruit capacity for production of 2-methylbutyl acetate, 2-methylpropyl acetate, hexyl 2-methylbutanoate, pentyl hexanoate, 2-methylbutyl 2-methylbutanoate, butyl 2-methylbutanoate, and butyl hexanoate in ‘Fuji’ apples after ultra-low oxygen storage^11,38^. Selected metabolites identified in the above-mentioned apple cultivars, and for which maturity-dependent up-regulation was associated with enhanced LOX and HPL activities, were also synthesized in higher capacities for more mature ‘Honeycrisp’ apples. Hexanal, a metabolite found upregulated in the current study as a result of the fruit ripening process in ‘Honeycrisp’ apples, is produced from hydroperoxides of the unsaturated fatty acids, while its biosynthesis is governed by the action of hydroperoxide lyase^29,37^. The accumulation of fatty acids in ‘Greensleeves’ apples during ripening was concomitant with increases in total aldehydes^11^; hence, upregulation of hexanal in more ripe ‘Honeycrisp’ apples may be attributed to accumulation of linoleic acid. Conversely, hexanal content was reported to decrease with respect to the harvest date of ‘Bisbee’ apples, and hexanal concentration was decreased in ‘Starkspur Golden’ apples during growth, and from 33 to 145 days after full bloom^39^.

Branched chain esters are characteristic of the ‘Honeycrisp’ apple variety and many other cultivars^40^. In the current work, butyl 2-methylbutanoate, propyl 2-methylbutanoate, ethyl 2-methylbutanoate, pentyl 2-methylbutanoate, butyl 2-methylpropanoate, hexyl 2-methylbutanoate, and 2-methylbutyl acetate were found to be upregulated in advanced maturity fruits. Branched chain esters are produced through the isoleucine biosynthesis pathway and from the breakdown of the branched-chain amino acids isoleucine, leucine, valine, alanine and aspartic acid^11,12,29,36,37^. Isoleucine is an important precursor of 2-methylbutyl and 2-methylbutanoate esters, which were found differentially overexpressed in some apple cultivars presenting advanced maturity as a result of increased isoleucine quantities in apple fruit skin during ripening^11,40^.

In addition to esters, estragole, *trans*-anethole, *cis*-anethole, 1-octen-3-ol, hexanal, and (2*E*,4*E*)-2,4-hexadienal were found to be upregulated as a result of ripening in the present study. While the available literature associated with apple fruit does not designate 1-octen-3-ol as a biomarker of fruit development, production of this metabolite has been previously observed in ripening banana cultivars under cold storage conditions^41^. In mushrooms, biosynthesis of this metabolite has been attributed to oxygenation of linoleic acid and subsequent cleavage of the fatty acid hydroperoxide^42^. On the other hand, a higher abundance of 2,4-hexadienal was observed during storage of ‘Royal Gala’ apples under extremely low O_2_ level conditions^43^.

The phenylpropanoid pathway has been proposed for the synthesis of estragole, a metabolite found to be upregulated as a result of ripening of ‘Honeycrisp’ apples^37^. In a study performed by Schaffer *et al*., external ethylene application to a transgenic line of ‘Royal Gala’ apple resulted in increased production of ester, terpene, and phenylpropanoid volatile compounds, estragole being the metabolite representative of the phenylpropanoid pathway^37^. Benzenoid and phenylpropanoid volatile metabolites are primarily derived from phenylalanine via the β-oxidative pathway, although they can be formed via non-oxidative reactions as well^44^. Schaffer *et al*. have demonstrated that selected genes associated with ethylene-induced ripening and the production of a defense ripening response are ethylene up-regulated, indicating that production of associated volatile defense chemicals is triggered by fruit ripening^37^. Fruit ripening, in turn, has been associated with increased susceptibility to pathogens, with ripe fleshy fruit previously shown to be more susceptible to decomposition and disease than unripe fruit^45^. In the current work, the metabolomic fingerprint of higher maturity ‘Honeycrisp’ apples captured by *in vivo* DI-SPME indicated up-regulation of phenylpropanoid metabolites, thus providing further evidence that ripening triggers defense mechanisms and biosynthesis of bioactive compounds that are involved in plant defense^37^.

In contradiction to other studies that reported enhanced biosynthesis of α-farnesene during fruit ripening, the metabolite profile of more mature ‘Honeycrisp’ apples was not characterized by up-regulation of this compound, nor enhanced production of metabolites originating from the α-farnesene synthesis pathway^12,37,46^. Oxidation of α-farnesene in peel tissues has been reported to be associated with superficial scald development, with levels of this metabolite reported to increase with apple ripening in both scald-resistant and scald-susceptible cultivars^46,47^. The development of superficial scald is also characterized by enhanced production of α-farnesene oxidation products, including conjugated trienols, 6-methyl-5-hepten-2-one, and 6-methyl-5-hepten-2-ol. As shown in Supplementary Fig. 7, the production of 6-methyl-5-hepten-2-one was enhanced in more mature ‘Honeycrisp’ apples as a result of fruit ripening. Considering the feasibility of *in vivo* SPME to detect metabolic alterations caused by fruit ripening, the technique could be useful in precociously identifying metabolic changes associated with development of scald disorders, such as soft scald in ‘Honeycrisp’ apples prior to the visual onset of the disorder^48^.

In addition to the above discussed metabolites, which were found to be upregulated in ripe fruit, two unknown metabolites were found to be upregulated in fruits with lower maturity index (see Supplementary Table 1). Examinations of EI mass spectra and GCxGC elution coordinates (see Supplementary Fig. 8) combined with a thorough literature review provided evidence that these two metabolites are Amaryllidaceae alkaloids of the lycorenine‐type^49^. Proposed fragmentation scheme and details associated with tentative analyte assignment are given in Supplementary Fig. 9.

Amaryllidaceae alkaloids are apolar plant metabolites originating from the precursor O-methylnorbelladine, which is synthesized from the amino acids phenylalanine and tyrosine^50^. Amaryllidaceae alkaloids, in particular, have a wide range of biological activities, including anti-cancer, anti-viral, anti-parasitic, and acetylcholinesterase (AChE) inhibitory activity, the latter playing a crucial role in the treatment of Alzheimer’s disease^50–52^. However, Amaryllidaceae alkaloids are characteristic of the Amaryllidaceae plant family and their presence has not yet been confirmed in fruits^50^. However, a few reports of detecting Amaryllidaceae alkaloids in species not belonging to the Amaryllidaceae have been published^53,54^. The unexpected occurrence of these metabolites in species not belonging to the Amaryllidaceae has been attributed to convergent evolution, which is a frequent phenomenon occurring in plant specialized metabolism and that has been linked to alkaloid biosynthetic pathways^53^. The correlation between alkaloid biosynthesis and fruit development and ripening has been studied in tomato cultivars by Eltayeb *et al*.^55,56^. The authors reported that lower tomatine levels were observed in cultivars with accelerated fruit ripening^55^. As a result of detecting changes in metabolic profiles induced by differences in fruit maturity and new metabolites affected by maturity stage of apple fruit, the results obtained in the current study illustrate that *in vivo* DI-SPME combined with mass spectrometric techniques is a powerful metabolic profiling approach with potential to be used as a tool for discovery of new natural bioactive molecules such as Amaryllidaceae alkaloids and other metabolites with potential AChE inhibitory activity.

Several metabolites were found unique to the *in vivo* DI-SPME metabolic fingerprint as compared to *ex vivo* DI-SPME for the same apple samples (Fig. 3, Supplementary Table 3). The annotation of identity for 1,4-diacetylbenzene was accomplished by mass spectral library searching, comparison between experimental and literature RI, and injection of reference standards. Tikunov *et al*. reported that the biosynthetic pathway for these acetophenone derivatives is still unclear in their large-scale HS-SPME profiling and comparative multivariate analysis platform for tomato samples^57^. In a metabolite-metabolite correlation matrix composed of 322 compounds from tomato samples, 4-methylacetophenone and acetophenone were observed to cluster with terpenoids and cyclic carotenoid volatiles, respectively^57^. For Analyte 1, the mass spectral library searching and proposed fragmentation procedure (see Supplementary Fig. 10), corresponded to 1-hepten-4-ol, however, there was disagreement between experimental and expected RI values and GCxGC elution coordinates. According to the GCxGC peak apex plot, the retention time coordinates of this metabolite were close to those obtained by saturated carboxylic acids; however, the EI mass spectrum of the unknown metabolite did not correspond to the fragmentation procedure expected for carboxylic acids.

The presence of unique metabolites in the *in vivo* metabolic fingerprint is expected, since during traditional approaches of sample preparation, the harvesting process itself can lead to significant metabolome perturbations, including enzymatic degradation and oxidation resulted by wounding of the plant^58^. Freezing in liquid nitrogen may result in degradation of metabolites, emission of touch- or wound-induced metabolites, and non-reversible loss of metabolites by absorption to cell walls^5,6^. Metabolite decomposition and interconversion following fruit disruption have been reported in determinations of flavour compounds and pyrophosphate levels in plants^4,59,60^. Differing metabolic profiles corresponding to intact versus disrupted leaves have been observed as well; in the latter scenario, destruction of tissue compartmentalization releases hydrolytic enzymes that are responsible for a number of reactions^2^.

A common feature among all compounds found to be specific to the profile obtained via *ex vivo* sampling is their biosynthesis from polyunsaturated fatty acids, including oleic acid, linoleic acid, and linolenic acid^61^. Their formation is the result of lipid peroxidation, which is known to be an important oxidative degradation mechanism in food commodities^61^. Contreras and Beaudry investigated differences in production of LOX-associated apple volatiles by intact and disrupted apple tissues throughout maturation^62^. The presence of *cis*-3-hexenal, *trans*-2-hexenal, *cis*-3-hexenol, *trans*-2-hexenol, *cis*-3-hexenyl acetate, and *trans*-2-hexenyl acetate was detected only in the disrupted system, leading to the conclusion that these volatiles are dependent on the action of LOX pathway enzymes after tissue disruption. Riley and Thompson observed that both ripe and unripe intact tomato fruit contained minimal endogenous aldehyde content^63^; however, following homogenization of tomato fruit in the absence of buffer, aldehyde levels increased rapidly in ripe fruit, whereas the aldehyde-generating capacity in green fruit was not significant^63^. In addition to suggesting that the metabolome profile and sample integrity are altered by the choice of sample preparation, these results indicate that the ability of a given metabolomics platform to discriminate between metabolic fingerprints of ripe and unripe fruit is significantly influenced by the employed sample preparation and extraction methods.

*In vivo* sampling also enabled detection of LOX-associated volatiles, including hexanal, *cis*-3-hexenal, *trans*-2-hexenal, *cis*-3-hexenol, *trans*-2-hexenol, *cis*-3-hexenyl acetate, and *trans*-2-hexenyl acetate in the metabolome of intact apple fruit (Supplementary Table 2). While the role of LOX in the volatile metabolite biosynthesizing capacity of intact systems is not clearly understood, our results are in accordance with the literature reporting the presence of hexenal and *trans*-2-hexenal in intact apple fruit, while contradictory to the hypothesis that the biosynthesis of these volatiles is triggered upon tissue disruption^11,64,65^. This is also further supported by previously presented data, which clearly illustrated that *in vivo* SPME analysis of intact plant tissues of more mature ‘Honeycrisp’ apples provided evidence of up-regulation of selected metabolites, including those that are known to be produced via the LOX pathway. Aiming to further substantiate that LOX-associated volatile production in intact ‘Honeycrisp’ apples results from enhanced lipid metabolism, rather than potential tissue perturbation during *in vivo* sampling, a comparative investigation was carried out. To this end, *ex vivo* HS-SPME sampling of intact apples, peeled apples, and apples pierced by SPME devices (resembling the sampling procedure used during actual *in vivo* sampling) was carried out for a period of time equivalent to the duration of actual *in vivo* experiments to capture any potential variations in volatile emissions among the three sampled systems. Several metabolites were monitored: hexyl acetate, a product of the LOX pathway; α-farnesene, a defense compound whose production is enhanced after disturbance of the apple tissue; and branched-chain esters, including hexyl 2-methylbutanoate, butyl 2-methylbutanoate, propyl 2-methylbutanoate, and ethyl 2-methylbutanoate, which were found statistically up-regulated in the more mature apple group. The results of this investigation yielded no statistical differences in the emission of these compounds between intact and punctured systems (Supplementary Fig. 11). On the other hand, as a result of the increased activity of LOX pathway enzymes after tissue disruption, hexyl acetate was found in larger quantities in volatile emissions generated after peel removal in comparison to intact and punctured apples. The findings therefore confirm the suitability of *in vivo* SPME as a non-invasive sampling tool capable of capturing the metabolic composition of the biological system without inducing metabolic alterations related to tissue wounding.

*In vivo* SPME provided a unique metabolic fingerprint despite ensuring that a strict metabolism quenching protocol was followed during sample preparation prior to *ex vivo* extraction. Furthermore, the occurrence of metabolites known to be volatile end-products of lipid peroxidation in the *ex vivo* metabolic profiles (Supplementary Fig. 5) suggests that rapid degradation of metabolome integrity may be encountered in metabolomics studies that employ traditional methods of sample preparation. The results of the current work further corroborate that unlike traditional approaches, non-exhaustive extraction and the miniaturized format of the SPME device allow *in vivo* sampling of living systems with minimum perturbation. Furthermore, the presence of LOX-derived metabolites in intact fruit sampled by *in vivo* SPME was demonstrated to be associated with increased lipid metabolism during ripening rather than fruit perturbation. Based on literature studies, LOX influence on biosynthesis of volatiles in intact fruit is still not known and is considered negligible; hence, future implementations of *in vivo* SPME for sampling both volatile and thermally labile metabolites may provide further evidence with respect to the activity and biological role of this widely studied plant enzyme.

## Methods

### Analytical supplies and reagents

Acetone (HPLC grade) and methanol (HPLC grade) were obtained from Caledon Laboratories (Georgetown, ON, Canada). Water samples spiked with metabolite standards (Sigma–Aldrich, Oakville, ON, Canada) were analyzed by HS-SPME to establish the system precision procedure and GCxGC, mass spectral and linear temperature-programmed retention index (RI) databases^34,66^. An automated SPME holder and 10 mL amber screw cap vials were purchased from Supelco (Oakville, ON, Canada).

### *In vivo* DI-SPME sampling

As per our previous experiments, 50/30 μm divinylbenzene/carboxen/polydimethylsiloxane (DVB/CAR/PDMS) fiber assemblies (automated format, stableflex, 23-gauge needle size) (Supelco, Oakville, ON, Canada) were used for all experiments^66,67^. SPME fiber coatings were conditioned as per the supplier recommendations. Prior to *in vivo* extraction, additional fiber conditioning was performed for 5 min at 270 °C, followed by sealing needles of SPME fiber assemblies with Teflon caps. Fiber coatings were exposed into fruit tissue from directions that were perpendicular with respect to the fruit stem. As per the results of a previously published study requiring determination of analytical precision for different *in vivo* sampling designs, the inserted coatings were kept at a close distance from each other (1.5 cm)^34^. Sampling depth and extraction time were 3 cm and 60 min, respectively. Triplicate *in vivo* determinations were performed for each apple. Following extraction, coatings were wiped with Kim Wipes and washed in water for 10 s, then again wiped with Kim Wipes prior to withdrawal into their respective needles. During transportation, SPME fiber assemblies were stored in dry ice at −70 °C. For experiments focused on a comparison of metabolite coverage between *in vivo* and *ex vivo* modes of extraction, desorption was performed immediately after arrival to the laboratory. Alternatively, during long sample sequences, fiber coatings were stored at −30 °C prior to analysis.

For analysis of fruit at two different maturity stages, sampling was performed in 2010 (temperature ranged from 24 °C to 21 °C). Five apples of earlier maturity index (HC-O apples, codes 1–5) and 5 apples of later harvest maturity (HC-L apples, codes 1–5) were sampled in total. Earlier maturity fruit was characterized by the following attributes: 7 starch index (based on Cornell starch chart, 1–8 scale (8 = no starch)), 40–60 ppm internal ethylene concentration, 14–15 lb firmness, average 12.4% soluble solids, 570 mg malic acid per 100 mL juice, 70–80% red blush with yellow-green background color. For later harvest maturity, attributes were as follows: 8 starch index (based on Cornell starch chart, 1–8 scale (8 = no starch)), 20–40 ppm internal ethylene concentration, 13–14 lb firmness, average 12.9% soluble solids, 520 mg malic acid per 100 mL juice, 80–90% red blush with yellow background color.

The purpose of the *in vivo* sampling conducted in 2011 (temperature was 18 °C) was to make a comparison of metabolite coverage obtained with *ex vivo* and *in vivo* SPME.

### *Ex vivo* SPME samples and sample preparation procedure

‘Honeycrisp’ apples (diameter of approximately 6–7 cm) were harvested after *in vivo* sampling from a commercial orchard in Simcoe (Norfolk County, ON, Canada). Fruit were immersed in liquid nitrogen after harvesting and stored in dry ice at −70 °C during transportation. Individual apples were rinsed with distilled water, dried with Kim Wipe and sliced in random positions. Frozen apple tissue (100 g) was homogenized for 1.5 min in 250 mL of saturated sodium chloride solution. Following addition of 250 mL of nanopure water, samples were homogenized for an additional 1 min. The final homogenate was transferred into 20 mL vials and protected from light. Samples were stored in freezer at −30 °C until analysis. For comparisons of metabolite coverage between *ex vivo* and *in vivo* modes, where possible, samples were analyzed immediately after homogenization so as to eliminate freezing and thawing sample preparation steps. For the majority of experiments requiring high throughput, vials containing homogenate were thawed for 20 min individually in a temperature-controlled water bath maintained at 30 °C. 10 mL portions of thawed homogenate were transferred into 10 mL screw-cap amber vials for DI-SPME extraction. Extraction parameters throughout the study entailed an incubation time of 5 min followed by 60 min of extraction at 30 °C and 500 rpm. SPME extraction was performed immediately after thawing of samples in order to avoid storage on the autosampler tray. After DI-SPME extraction, SPME extraction phase was immersed in 10 mL of nanopure water prior to desorption in order to remove non-volatile interferences from the coating surface.

In order to determine retention indices in the first dimension and monitor GCxGC-ToFMS system performance, HS-SPME extraction of water samples spiked with 52 metabolites belonging to various chemical groups frequently encountered in plants (*n*-alkanes (C_8_-C_19_), aldehydes, 2-ketones, ethyl esters, monoterpenes (hydrocarbons, ketones, aldehydes, oxides and alcohols), sesquiterpenes (hydrocarbons, alcohols), 1-alcohols, and 2-alcohols) was carried out using the same extraction conditions as per the procedure outlined above (preparation of spiked water standards was carried out as per the procedure in refs. ^34,66^). In addition, for QC system checks, 3 mL portions of combined homogenate from individual apples were transferred to 10 mL vials and analyzed by HS-SPME, using the same extraction conditions as described above for DI-SPME analyses of apple samples.

### SPME sampling of volatile emissions

A dedicated sampling chamber was designed (Supplementary Fig. 12) for *ex-vivo* HS-SPME sampling of apple volatile emissions. The sampling chamber was sealed with a hermetic lid in order to avoid losses of emitted volatile compounds. The lid of the chamber was modified to allow simultaneous sampling with three SPME devices through gas-tight septa. The SPME coating used for extraction was DVB/CAR/PDMS (Supelco, Oakville, ON, Canada). After sealing the chamber containing the fruit, an incubation time of 60 min was observed to ensure headspace enrichment of volatile compounds emitted by the apple. SPME fibers were then inserted into the chamber for extraction for a period of 60 min at 20 (±1) °C. Following extraction, fibers were sealed by Teflon caps and stored in dry ice at -70 °C until desorption. Three different sets of experiments were carried out. For set #1, an intact apple was placed in the chamber, and sampling was carried out simultaneously by three SPME fibers according to the abovementioned parameters. For set #2, aiming to reproduce the conditions of *in vivo* sampling, an apple was pierced with SPME fibers positioned as per the procedure described in the previous section, “*In vivo DI-SPME sampling”*, followed by placement of the fruit in the chamber, incubation, and extraction. For set #3, the abovementioned sampling procedure was applied to sample an apple after removal of its peel.

### GCxGC-ToFMS analysis and data processing

A LECO Pegasus 4D GCxGC-ToFMS instrument equipped with the Agilent 6890 N GC and a high speed ToF mass spectrometer (LECO, St. Joseph, MI, USA) was used for data acquisition. Modulation was performed with a dual-stage quad-jet cryogenic modulator (licensed from Zoex, Houston, TX, USA). A MultiPurpose Sampler (MPS 2) autosampler (Gerstel GmbH, Mulheim an der Ruhr, Germany) was used for automation of the SPME process. The first dimension column was a 5% phenyl 95% dimethylpolysiloxane Rxi-5SilMS (30 m × 0.25 mm ID × 0.25 µm) capillary column (Restek, Bellefonte, PA, USA). In the second dimension, polar polyethylene glycol columns, including BP 20 (SGE Incorporated, Austin, TX, USA) and Stabilwax (Restek, Bellefonte, PA, USA) (Stabilwax 1 m × 0.25 mm ID × 0.25 µm column, BP 20 1.11 m × 0.10 mm ID × 0.10 µm column) were used.

The GC inlet was equipped with a high-pressure Merlin Microseal septumless injection system (Merlin Instrument Co., Half Moon Bay, CA, USA) and a 0.75 mm ID narrow-bore liner from Supelco (Oakville, ON, Canada). Desorption was carried out at 270 °C. For analysis of apple samples of differing maturities, the Stabilwax column was used in the second dimension. Helium was used as a carrier gas with a flow rate of 2.0 mL/min. The primary dimension oven temperature programming was set to 40 °C (5 min hold time), followed by 5 °C/min rate to 235 °C (10 min hold time). The secondary oven temperature programming was equivalent except for the 20 °C temperature offset above the primary oven temperature. The modulation parameters consisted of using a modulator temperature offset of 25 °C and a 3.5 s modulation period (0.7 s hot pulse time, 1.05 s cool time). The acquisition rate was 200 spectra/sec.

For experiments aimed at a comparison of *ex vivo* and *in vivo* metabolome coverage, a BP 20 column was used in the second dimension. Helium was used as a carrier gas with a flow rate of 1.5 mL/min. The primary dimension oven temperature programming was set to 40 °C (5 min hold time) and then to 250 °C at a rate of 5 °C/min (10 min hold time). The secondary oven programming was equivalent, except for a 10 °C temperature offset above the primary oven temperature. Modulation was carried out using a modulator temperature offset of 30 °C, and a 4 s modulation period (0.8 s hot pulse time, 1.20 s cool time). An acquisition rate of 250 spectra/s was employed.

For all studies, the transfer line and ion source temperatures were set to 240 and 220 °C, respectively. The mass spectrometer was operated in electron ionization (EI) mode with a mass acquisition range of 33–550 amu. Data acquisition and processing were performed with ChromaTOF (version 4.24) software. Library searching was carried out in the National Institute of Standards and Technology (NIST, version 2.05), Terpene, and Wiley 8 mass spectral databases.

Data processing consisted of several steps. After processing of raw data, mass spectral deconvolution and second dimension peak combination, peaks/features that met a certain mass spectral similarity threshold (not less than 700) were preserved. For statistical data analysis aimed at differentiating between metabolome corresponding to fruit with different maturities, the sample for which the highest number of peaks was obtained was used as a reference for generation of a data matrix. Manual processing was conducted in order to eliminate blank peaks, peaks for which separation efficiency and modulator effectiveness were not optimum, and peaks with overloaded and tailing peak profiles. In total, 225 true metabolites were submitted to the automated ‘compare-to-reference’ ChromaTOF software alignment procedure. The automated data processing procedure was inspected to ensure proper unique mass assignment, metabolite alignment, second dimension peak combination into one-dimensional peak entries, and second dimension peak integration. Metabolite identification was performed on the basis of retention time and mass spectral comparison with reference standards, retention index comparison, and structured GCxGC separations.

### GC-ToFMS analysis of volatile apple fruit emissions

A LECO Pegasus 4D GC-ToFMS instrument equipped with an Agilent 6890 N GC and a high speed ToF mass spectrometer (LECO, St. Joseph, MI, USA) was used for data acquisition. The chromatographic column was a SLB-IL60 (30 m × 0.25 mm ID × 0.2 μm) capillary column (Supelco, Bellefonte, PA, USA). A GC inlet was equipped with a 0.75 mm ID narrow-bore deactivated Sky® liner (Restek, Bellefonte, PA, USA). Desorption of SPME fibers was carried out at 270 °C for 15 min. Helium was used as a carrier gas with a flow rate of 1.5 mL/min. Oven temperature was set to 35 °C for 5 min, to 100 °C at 15 °C/min (5 min hold), to 260 °C at 15 °C/min (6 min hold). Transfer line and ion source temperatures were set to 200 and 260 °C, respectively. The acquisition range was set to 35–550 *u*, electron ionization was enabled, and the acquisition rate was 20 spectra/sec. Data acquisition and processing were performed with ChromaTOF (version 4.5) software.

## Supplementary information

Supplementary Information.
