# Supplementary material for: Application of in vivo solid phase microextraction (SPME) in capturing metabolome of apple (Malus ×domestica Borkh.) fruit
Source: Sci Rep. 2020 Apr 21;10:6724. doi: 10.1038/s41598-020-63817-8 (PMC7174353; doi:10.1038/s41598-020-63817-8)
Supplement: Supplementary file 1 — Supplementary Information. [file 41598_2020_63817_MOESM1_ESM.pdf]

Application of *in vivo* solid phase microextraction (SPME) in capturing metabolome of apple (*Malus* ×*domestica* Borkh.) fruit

Sanja Risticevic[1], Erica A. Souza-Silva[1,2], Emanuela Gionfriddo[1,3], Jennifer R. DeEll[4], Jack Cochran[5], W. Scott Hopkins[1], Janusz Pawliszyn[1*]

[1] Department of Chemistry, University of Waterloo, 200 University Avenue West, N2L 3G1, Waterloo, Ontario, Canada

[2] Departamento de Química, Universidade Federal de São Paulo (UNIFESP), Rua Prof. Arthur Riedel 275, 09972-270, Diadema, SP, Brazil

[3] Department of Chemistry and Biochemistry, University of Toledo, 2801 W. Bancroft St., 43606-3390, Toledo, Ohio, U.S.A.

[4] Ontario Ministry of Agriculture, Food and Rural Affairs, 1283 Blueline Rd. at Hwy #3, Box 587, N3Y 4N5, Simcoe, Ontario, Canada

[5] VUV Analytics, 715 Discovery Blvd, Ste 502, 78613, Cedar Park, Texas, U.S.A.

[*] corresponding author:

email: janusz@uwaterloo.ca

address: Department of Chemistry, University of Waterloo, 200 University Avenue West, N2L 3G1, Waterloo, Ontario, Canada

telephone: +1 519-888-4641

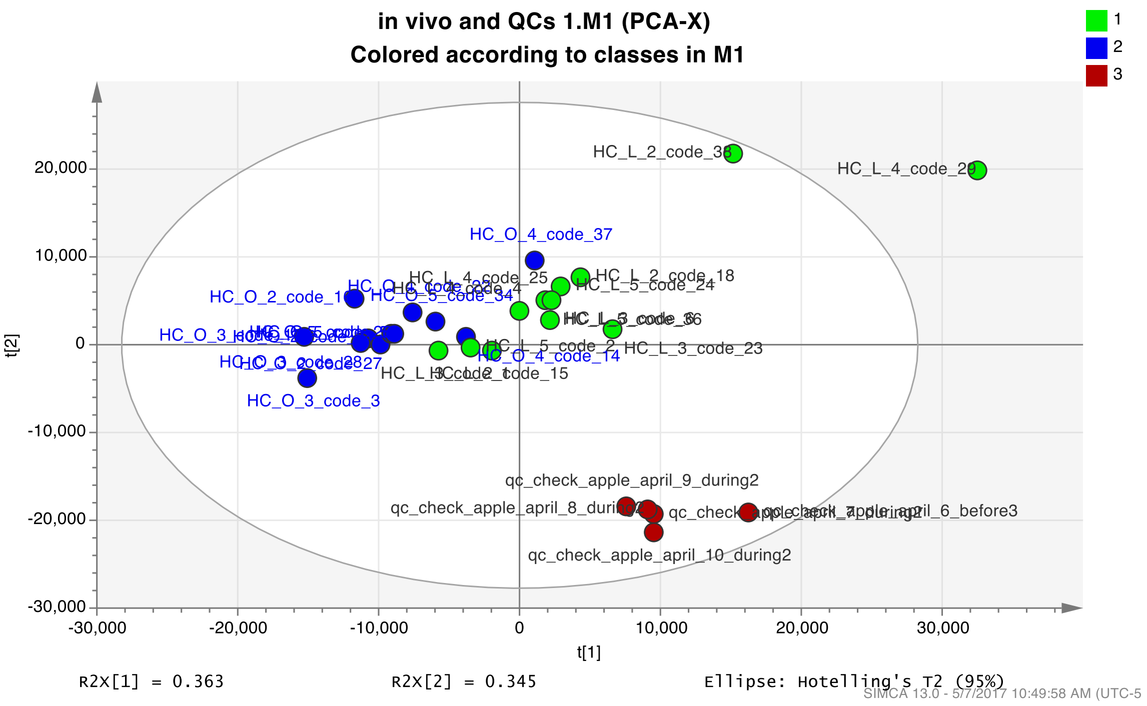

Figure 1. PCA scores plot of *in vivo* DI-SPME data for HC-L apples (higher maturity index, represented by green circles), HC-O apples (lower maturity index, represented by blue circles) and *ex vivo* HS-SPME data obtained for control samples (red circles).

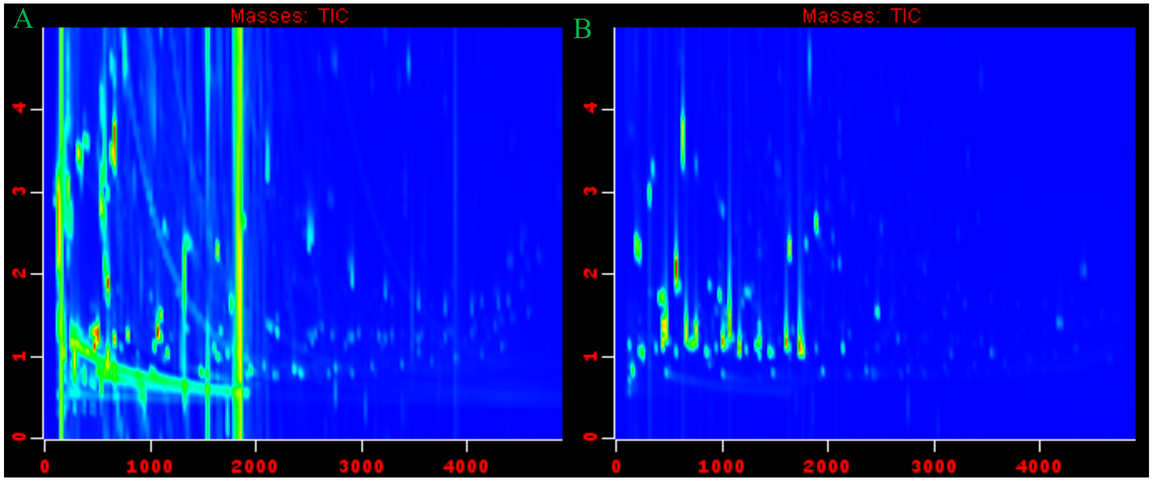

Figure 2. Contour plots of GCxGC-ToFMS TIC chromatograms corresponding to A – *in vivo* DI-SPME sampling and B – *ex vivo* DI-SPME sampling

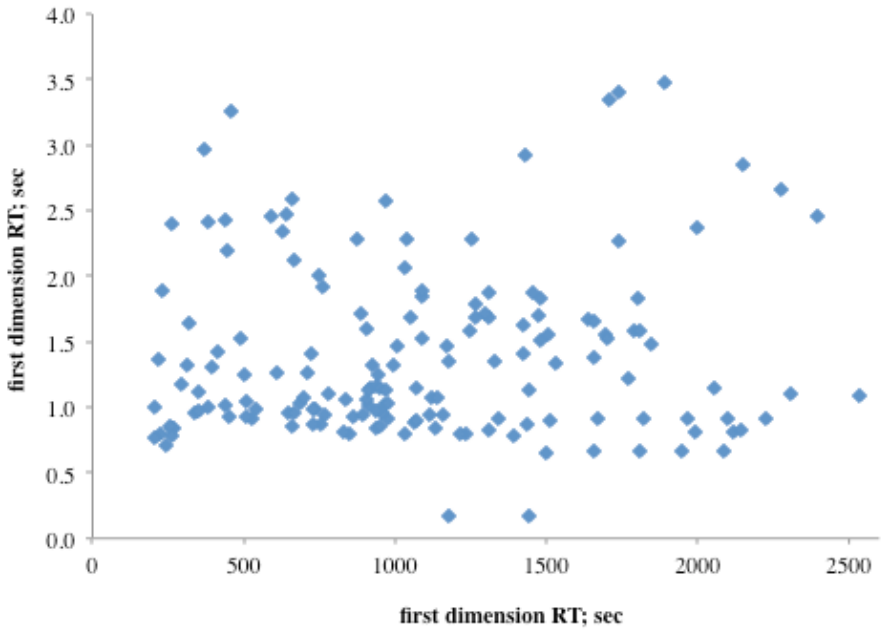

Figure 3. Tentatively identified metabolites (mass spectral similarity threshold 750) extracted by *in vivo* DI-SPME of apples, grouped in respective homologous compound series (sampling season 2011)

A

Peak True - sample "DVBCARPDMS_code_one_1", peak 5609, at 1760 , 3.496 sec , sec

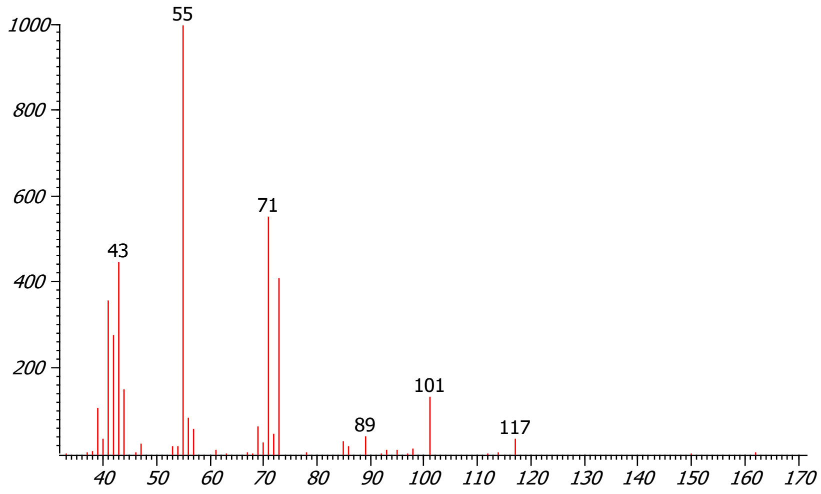

Peak True - sample "DVBCARPDMS_code_one_1", peak 6998, at 2280 , 2.172 sec , sec

B

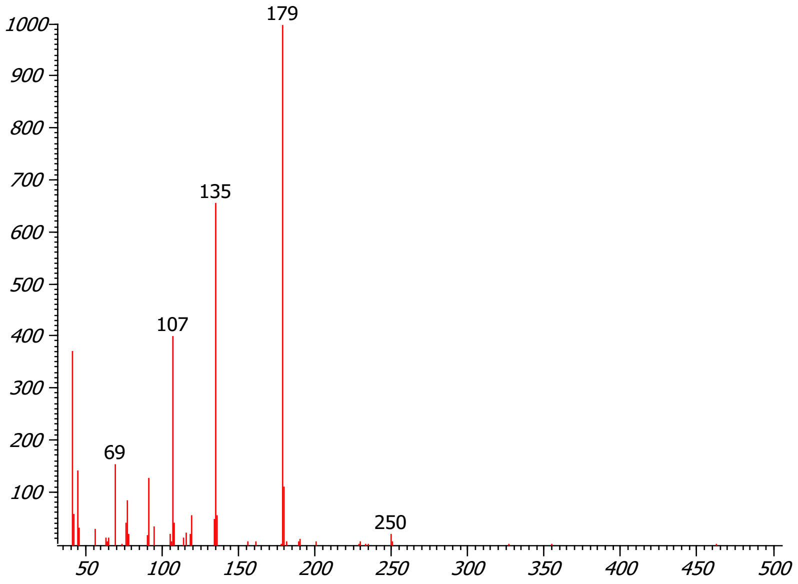

C

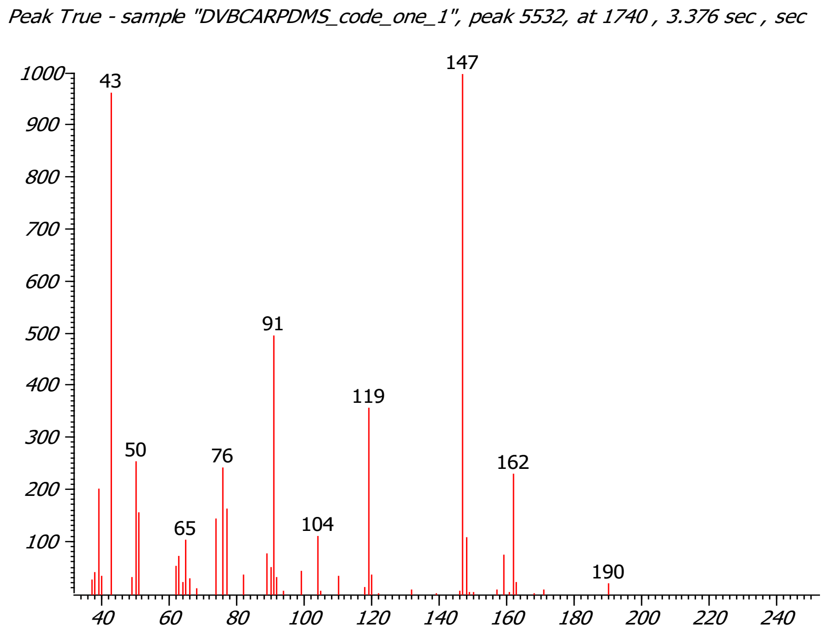

Figure 4. Mass spectra of analytes unique to *in vivo* DI-SPME sampling approach. A – unidentified analyte 1; B – unidentified analyte 2; C – 1,4-Diacetylbenzene (see Table 3).

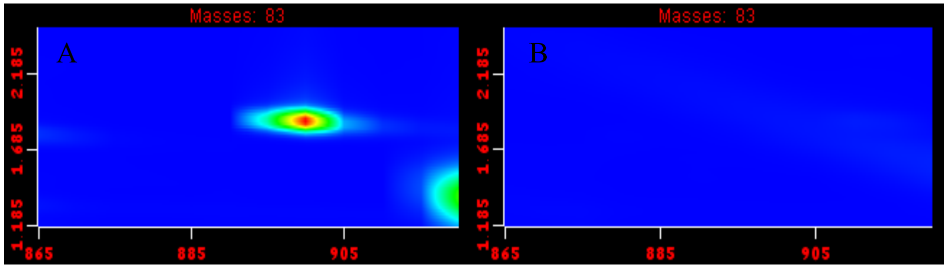

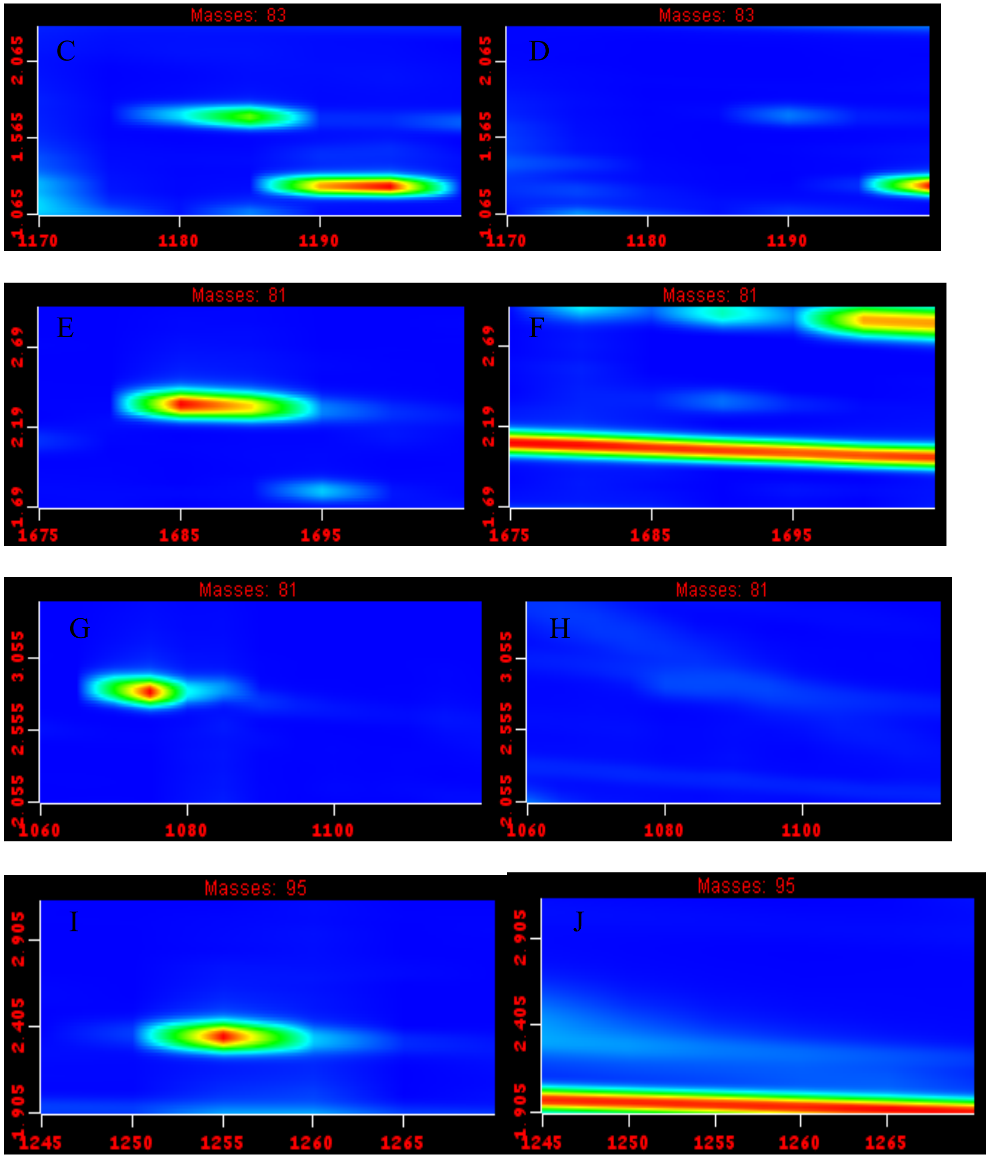
Masses: 83
C
2.065
1.565
1.065
1170
1180
1190
Masses: 83
D
2.065
1.565
1.065
1170
1180
1190
Masses: 81
E
2.69
2.19
1.69
1675
1685
1695
Masses: 81
F
2.69
2.19
1.69
1675
1685
1695
Masses: 81
G
3.055
2.555
2.055
1060
1080
1100
Masses: 81
H
3.055
2.555
2.055
1060
1080
1100
Masses: 95
I
2.905
2.405
1.905
1245
1250
1255
1260
1265
Masses: 95
J
2.905
2.405
1.905
1245
1250
1255
1260
1265

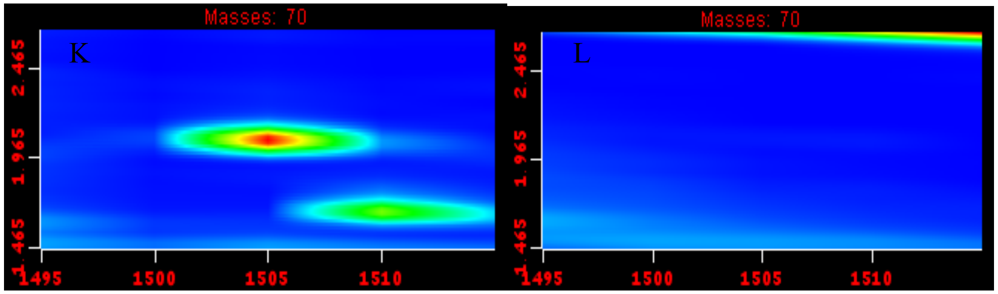

Figure 5. GCxGC extracted ion chromatograms corresponding to elution windows of metabolites unique to *ex vivo* approach. Chromatograms on the left represent *ex vivo* profiles and chromatograms on the right represent profiles obtained *in vivo*. A and B - (2E)-2-heptenal, C and D - (2Z)-2-octenal, E and F - (2E,4E)-2,4-nonadienal, G and H - (2E,4E)-2,4-heptadienal, I and J - (3E,5E)-3,5-octadien-2-one, K and L - (2E,6Z)-2,6-nonadienal.

A

B

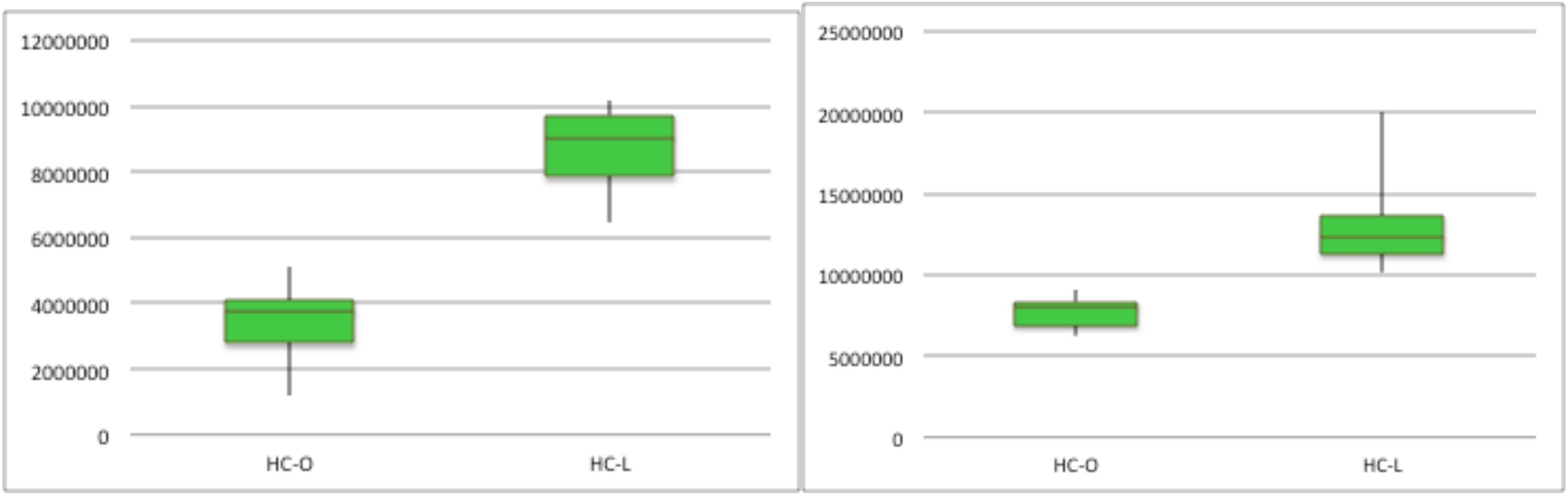

C D

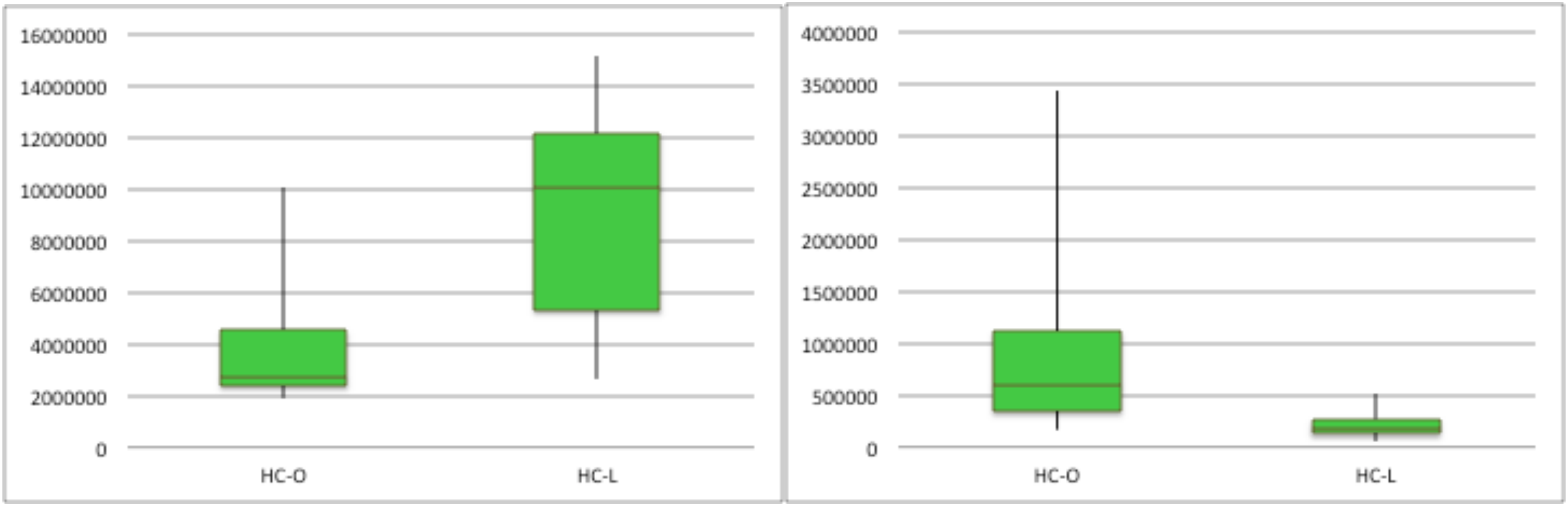

Figure 6. Box plots for A - butyl 2-methylbutanoate, B - estragole, C - hexanal, D - unknown # 1 (refer to Table 1) for metabolites upregulated and downregulated in 'Honeycrisp' apples as a result of maturity.

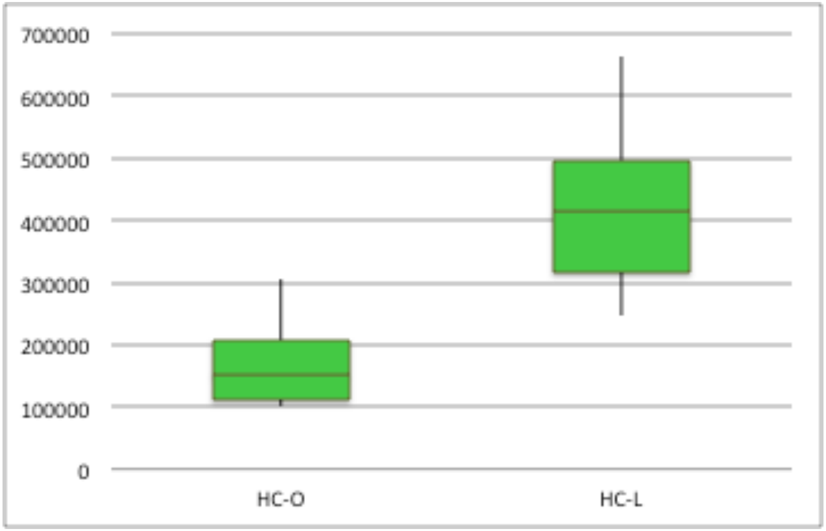

Figure 7. Box plot for 6-methyl-5-hepten-2-one, oxidation product of α-farnesene in less mature (HC-O) and more mature (HC-L) ‘Honeycrisp’ apples.

A

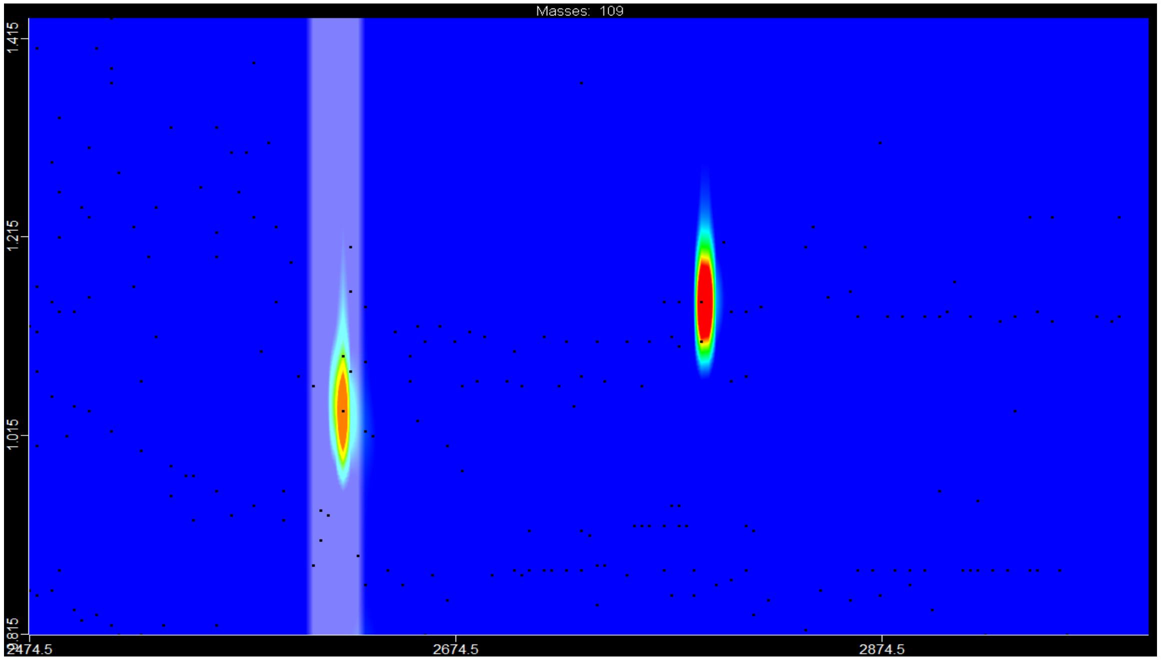

B

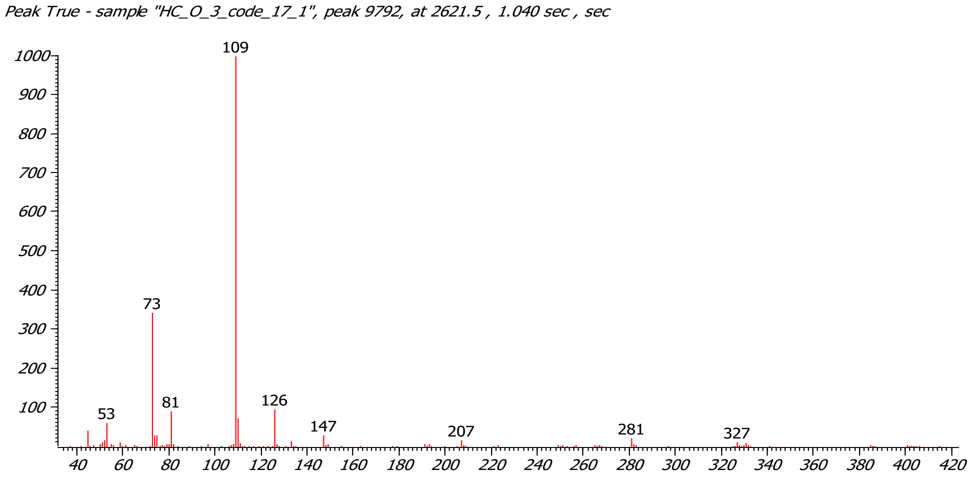
Peak True - sample "HC_O_3_code_17_1", peak 9792, at 2621.5 , 1.040 sec , sec
109
73
53
81
126
147
207
281
327

C

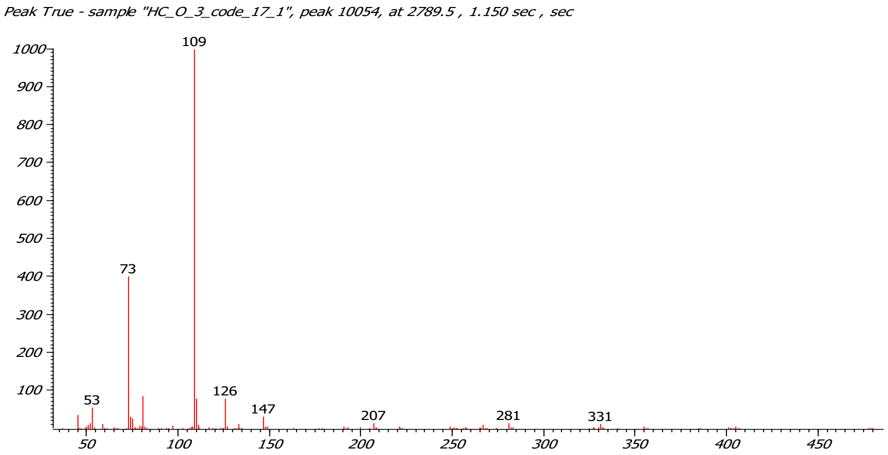
Peak True - sample "HC_O_3_code_17_1", peak 10054, at 2789.5 , 1.150 sec , sec
109
73
53
126
147
207
281
331

Figure 8. GCxGC extracted ion chromatogram (plot A) for two unknown metabolites upregulated in ‘Honeycrisp’ apples of lower maturity. EI mass spectra for unknown #1 (plot B) and unknown #2 (plot C) metabolites upregulated in ‘Honeycrisp’ apples of lower maturity (refer to Table 1), tentatively identified as Amaryllidaceae alkaloids of the Lycorenine-type.

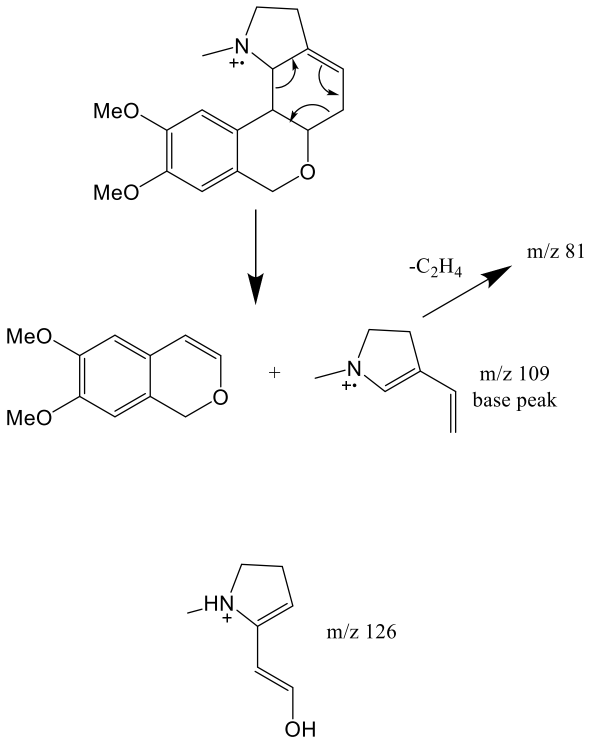

Figure 9. Fragmentation scheme for unknown #1 and unknown #2 (see Table 1) metabolites upregulated in ‘Honeycrisp’ apples of lower maturity. Analytes were tentatively identified as Amaryllidaceae alkaloids of the Lycorenine-type based on findings from Ibuka et al. (*Tetrahedron Lett.*, **1966**, No. 39, Pgs. 4745-4748). The base peak at m/z 109 arises from a retro Diels-Alder process, and this fragment can subsequently eliminate ethene to generate the fragment at m/z 81. The fragment at m/z 73 is a common ion – $C_3H_7OCH_2$, C_3H_7CHOH, or $C_2H_5OCHCH_3$ – and it is not especially diagnostic. However, it is consistent with fragmentation of the oxygen-containing (?isochroman?) ring system. The fragment at m/z 126 is consistent with addition of an OH group to the m/z 109 species.

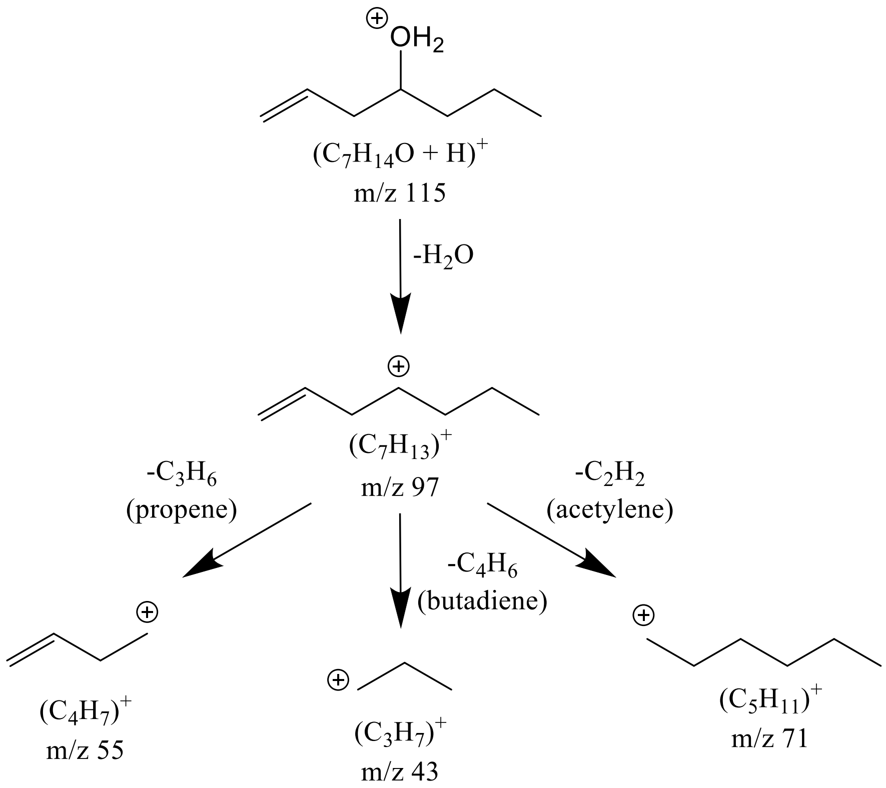

Figure 10. Fragmentation scheme for unidentified analyte 1 (library searching hit # 1: 1-hepten-4-ol, refer to Table 3). The dominant features at m/z 71, 55, 43 can been assigned to $C_5H_{11}^+$ (m/z 71), $C_4H_7^+$ (m/z 55), and $C_3H_7^+$ (m/z 43). The m/z 71 and 43 peaks are common fragments from alkyl chains. The m/z 55 peak suggests the presence of a double bond, since m/z 57 is expected for a saturated chain.

A

B

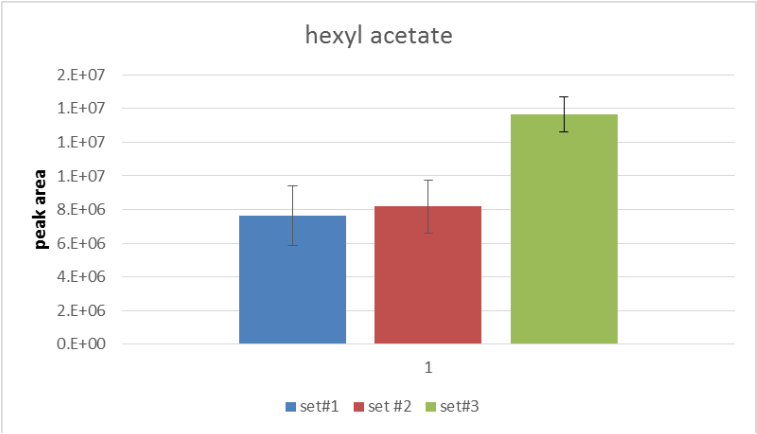
hexyl acetate
peak area
2.E+07
1.E+07
1.E+07
1.E+07
8.E+06
6.E+06
4.E+06
2.E+06
0.E+00
1
set#1
set #2
set#3

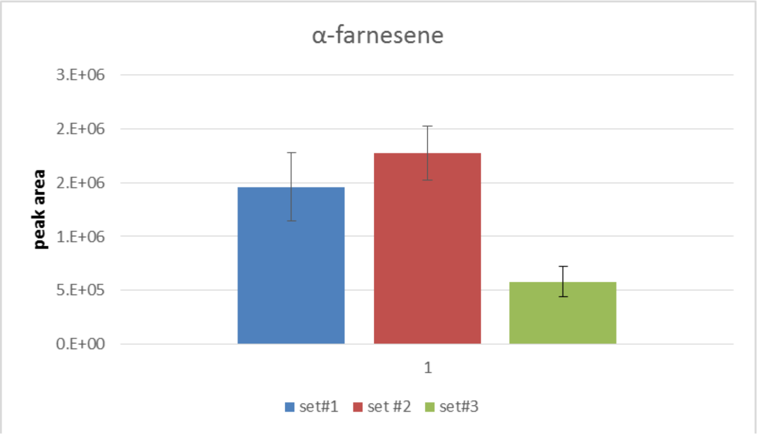
α-farnesene
peak area
3.E+06
2.E+06
2.E+06
1.E+06
5.E+05
0.E+00
1
set#1
set #2
set#3

C

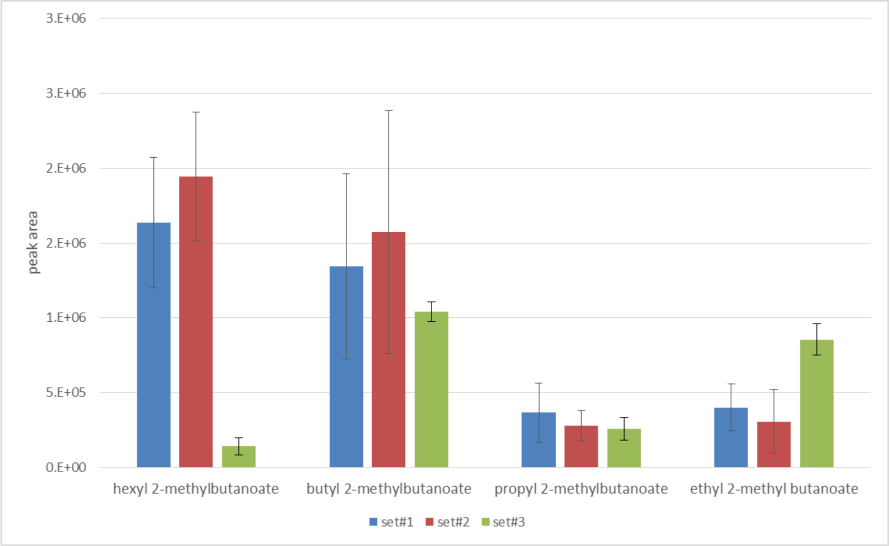
peak area
3.E+06
3.E+06
2.E+06
2.E+06
1.E+06
5.E+05
0.E+00
hexyl 2-methylbutanoate
butyl 2-methylbutanoate
propyl 2-methylbutanoate
ethyl 2-methyl butanoate
set#1
set#2
set#3

Figure 11. HS-SPME sampling performed on volatile emissions from apples under different experimental conditions: set #1 HS-SPME sampling of intact apple; set #2 HS-SPME sampling of apple pierced by SPME fibre (simulating *in vivo* sampling conditions); set #3 HS-SPME sampling of peeled apple.

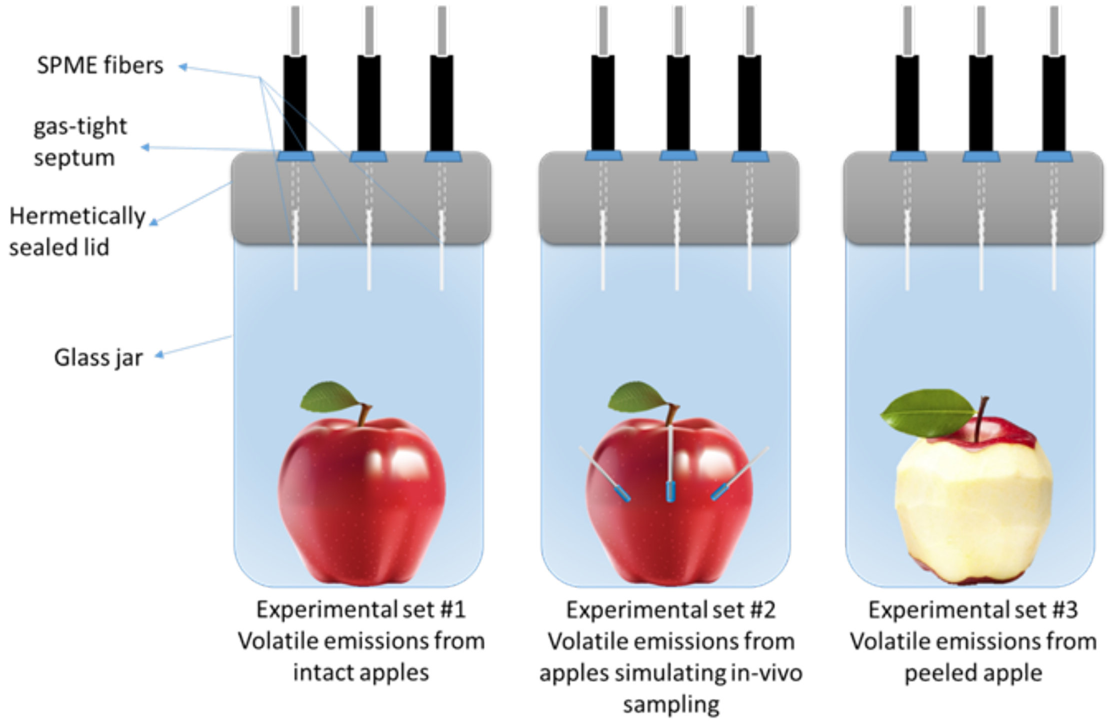

Figure 12. Experimental set-up for three experimental sets carried out during HS-SPME sampling of volatile apple emissions.

Table 1. Differentiating metabolites in analysis of 'Honeycrisp' apples of different maturities as determined by OPLS-DA analysis of *in vivo* DI-SPME data collected for 225 metabolites

Var ID (Primary)	analyte name	CAS #	$^{1}t_R$; sec	$^{2}t_R$; sec	RI_{exp}	RI_{lit}	unique mass	SIM	M4.p[1]	M4.p(corr)[1]
100	ethyl propanoate	105-37-3	161	0.71	724	717	102	810	0.11	0.9
17	1-propylethanoate	109-60-4	164.5	0.745	727	720	61	803	0.30	0.8
79	hexanal	66-25-1	276.5	1.045	805	801	56	940	0.16	0.6
55	ethyl butanoate	105-54-4	280	0.885	806	806	88	916	0.46	0.9
148	propyl propanoate	106-36-5	294	0.925	812	807	57	824	0.17	0.7
34	butyl acetate	123-86-4	308	1.02	818	819	73	901	0.39	0.6
53	2-methylethyl butyrate, ethyl 2-methylbutanoate	7452-79-1	385	0.83	852	842	102	804	0.27	0.9
8	2-methylbutyl acetate (1-butanol, 2-methyl-, acetate)	624-41-9	455	0.935	882	873	70	936	0.22	0.7
59	propyl butanoate	105-66-8	497	0.875	900	895	89	906	0.19	0.8
99	butyl propanoate	590-01-2	521.5	0.895	911	910	75	926	0.13	0.8
19	(2*E*,4*E*)-2,4-hexadienal (sorbic aldehyde)	4488-48-6	532	2.7	915	914	81	868	0.06	0.6
102	propyl 2-methylbutanoate	37064-20-3	605.5	0.79	948	946	103	905	0.16	0.9
98	butyl 2-methylpropanoate	97-87-0	623	0.785	955	953	89	900	0.07	0.9
131	1-octen-3-ol, 3-octenol	3391-86-4	682.5	2.12	982	978	57	949	0.05	0.7
54	butyl butanoate	109-21-7	717.5	0.85	997	999	89	945	0.15	0.8
80	ethyl hexanoate	123-66-0	724.5	0.875	1000	1003	88	918	0.22	0.9
209	5-hexene-1-ol, acetate; 5-hexenyl acetate	5048-26-0	731.5	1.16	1003	1001	54	861	0.05	0.7
60	butyl 2-methylbutanoate	15706-73-7	808.5	0.78	1041	1047	103	936	0.20	0.9
221	pentyl 2-methylbutanoate	68039-26-9	997.5	0.765	1137	1142	103	851	0.05	0.9
46	1-methoxy-4-(2-propenyl)benzene (4-allylanisole, estragole)	140-67-0	1106	1.71	1194	1201	148	957	0.19	0.8
81	hexyl 2-methylbutanoate	10032-15-2	1176	0.765	1235	1239	103	888	0.11	0.8

1	1-methoxy-4-(1*Z*)-1-propenyl-benzene (*cis*-anethole)	104-46-1	1204	1.8	1251	1253	148	955	0.06	0.7
2	(*E*)-1-methoxy-4-(1-propenyl)benzene (*trans*-anethole)	4180-23-8	1260	1.975	1284	1288	148	941	0.15	0.8
126	unknown #1	n/a	2621.5	1.035	2354	n/a	109	n/a	-0.06	-0.5
129	unknown #2	n/a	2789.5	1.15	2507	n/a	109	n/a	-0.07	-0.5

${}^{1}t_R$ – first dimension retention time
${}^{2}t_R$ – second dimension retention time
RI_{exp} – experimental linear temperature-programmed retention index
RI_{lit} – literature linear temperature-programmed retention index
SIM – mass spectral similarity

Table 2. Metabolites extracted by *in vivo* DI-SPME sampling of apples, grouped into homologous series (sampling season 2011). Tentative identification was performed on the basis of retention time and mass spectral comparison (mass spectral similarity threshold 750) with reference standards, retention index comparison, and GCxGC structured separations

analyte name/name of homologous series	**CAS #**	**${}^{1}t_R$; sec**	**${}^{2}t_R$; sec**	**RI_{exp}**	**RI_{lit}**	**unique mass**	**SIM**
Acetate Esters							
Ethyl Acetate	141-78-6	224	0.792	620	614	61	935
2-Propyl acetate	108-21-4	264	0.840	660	655	87	790
Propyl acetate	109-60-4	336	0.964	717	712	61	928
2-Methyl-1-propyl acetate (Isobutyl acetate)	110-19-0	452	0.928	777	782	56	930
Butyl acetate	123-86-4	540	0.992	819	819	73	938
2-Methylbutyl acetate	624-41-9	684	1.028	881	873	67	941
4-Penten-1-yl acetate	1576-85-8	696	1.072	886	890	68	872
Pentyl acetate	628-63-7	764	0.936	917	915	61	953
3-Methyl-2-buten-1-ol acetate (3-Methylbut-2-en-1-yl acetate, Prenyl acetate)	1191-16-8	780	1.100	924	920	67	810
(3Z)-3-Hexenyl acetate	3681-71-	912	1.016	985	998	67	931

	8						
(3Z)-3-Hexenyl acetate	3681-71-8	960	1.012	1008	1008	67	942
Hexyl acetate	142-92-7	976	0.916	1016	1012	84	894
(2E)-2-Hexenyl acetate	2497-18-9	976	1.036	1016	1019	67	927
Propanoate Esters							
Propyl propanoate	106-36-5	528	0.912	814	810	75	929
2-Methylpropyl propanoate	540-42-1	660	0.856	871	866	57	794
Butyl propanoate	590-01-2	752	0.872	911	910	87	949
Butyl 2-methylpropanoate	97-87-0	848	0.804	956	952	89	819
Hexyl 2-methylpropanoate (Hexyl isobutanoate)	2349-07-07	1232	0.796	1150	1150	89	898
Butanoate Esters							
Methyl butanoate	623-42-7	352	0.972	725	720	74	828
Ethyl butanoate	105-54-4	508	0.924	805	806	88	864
Propyl butanoate	105-66-8	728	0.864	900	895	71	846
Propyl 2-methylbutanoate	37064-20-3	832	0.808	948	946	103	853
Butyl butanoate	109-21-7	940	0.844	998	999	71	951
Butyl 2-methylbutanoate	15706-73-7	1032	0.800	1045	1047	103	888
Pentyl butanoate	540-18-1	1132	0.836	1096	1094	71	856
Pentyl 2-methylbutanoate	68039-26-9	1216	0.792	1141	1156	103	792
Hexyl butanoate	2639-63-6	1312	0.824	1193	1195	89	916
Hexyl 2-methylbutanoate	10032-15-2	1392	0.784	1240	1239	103	932
Hexanoate Esters							
Ethyl hexanoate	123-66-0	948	0.856	1002	1003	88	789
Aldehydes							
Butanal	123-72-8	204	0.768	600	596	72	839
2-Methylbutanal	96-17-3	268	0.840	664	658	58	904
Hexanal	66-25-1	508	1.044	805	801	56	852

Heptanal	111-71-7	736	0.984	904	906	58	779
Octanal	124-13-0	956	0.964	1006	1006	84	823
Nonanal	124-19-6	1156	0.940	1109	1107	57	922
Decanal	112-31-2	1340	0.920	1210	1208	57	892
Undecanal	112-44-7	1512	0.904	1313	1296	82	847
Dodecanal	112-54-9	1672	0.920	1413	1425	57	931
Tridecanal	10486-19-8	1824	0.916	1514	1519	82	na
Tetradecanal	124-25-4	1964	0.912	1615	1610	82	913
Pentadecanal	2765-11-9	2100	0.908	1719	1713	82	752
Hexadecanal	629-80-1	2228	0.912	1820	1811	82	938
2-Butenal	4170-30-3	240	0.704	636	640	70	808
(2E)-2-Methyl-2-butenal	1115-11-3	392	1.300	746	741	84	888
(2E)-2-Pentenal	1576-87-0	416	1.428	758	751	83	789
(2E)-2-Hexenal	6728-26-3	612	1.264	850	847	83	904
(2E)-2-Octenal	2363-89-5	1068	1.152	1063	1062	70	946
(3Z)-3-Hexenal	6789-80-6	504	1.248	803	800	69	897
(2E,4E)-2,4-Hexadienal (Sorbic aldehyde)	142-83-6	760	1.920	915	914	81	921
(2E,4E)-2,4-Octadienal	30361-28-5	1168	1.464	1115	1111	81	790
(2E,4E)-2,4-Decadienal	2363-88-4	1528	1.336	1323	1322	81	762
Ketones							
2,3-Butanedione	431-03-8	204	1.004	600	592	86	968
2-Pentanone	107-87-9	256	0.852	652	682	58	768
1-Penten-3-one	1629-58-9	296	1.180	692	683	55	885
2,3-Pentanedione	600-14-6	312	1.328	704	700	100	819
4-Methyl 2-pentanone (Methyl iso-butyl ketone)	108-10-1	384	0.996	742	733	100	852

Cyclopentanone	120-92-3	488	1.524	796	791	55	959
2-Cyclopenten-1-one	930-30-3	592	2.460	841	835	82	814
Cyclohexanone	108-94-1	720	1.412	897	901	98	898
2-Heptanone	110-43-0	732	0.988	902	893	58	829
1-Octen-3-one	4312-99-6	904	1.064	981	975	70	910
6-Methyl-5-hepten-2-one	110-93-0	916	1.140	987	986	108	822
Alcohols							
Methylbutenol	60766-00-9	220	1.368	616	611	71	898
2-Methyl-1-propanol	78-83-1	232	1.888	628	626	33	848
1-Butanol	71-36-3	264	2.404	660	662	56	951
3-Pentanol	584-02-1	320	1.644	708	693	59	928
3-Methyl-3-buten-1-ol	763-32-6	372	2.960	735	743	68	896
2-Methyl-1-butanol	137-32-6	384	2.420	742	731	53	909
1-Pentanol	71-41-0	440	2.428	771	775	42	927
3-Methyl-2-buten-1-ol	556-82-1	456	3.260	779	767	71	861
(3E)-3-Hexen-1-ol	928-97-2	628	2.336	857	856	67	846
(3Z)-3-Hexen-1-ol	928-96-1	640	2.476	862	866	67	922
(2E)-2-Hexen-1-ol	928-95-0	660	2.592	871	864	57	925
1-Hexanol	111-27-3	668	2.116	874	867	84	841
1-Heptanol	111-70-6	888	1.708	974	969	70	911
1-Octen-3-ol	53907-72-5	908	1.604	983	983	57	840
3-Octanol	589-98-0	944	1.252	1000	999	59	854
2-Ethylhexanol	104-76-7	1008	1.472	1033	1028	57	898
(3Z)-3-Octen-1-ol	20125-84-2	1048	1.688	1053	1054	67	828
1-Octanol	111-87-5	1092	1.520	1076	1074	70	882
(5Z)-5-Octen-1-ol	64275-73-6	1092	1.840	1076	1051	67	818
1,3-Octanediol	23433-05-8	1440	0.168	1269	1275	75	850
1-Dodecanol	112-53-8	1768	1.220	1476	1473	83	924
1-Tetradecanol	112-72-1	2052	1.148	1682	1676	83	876

1-Hexadecanol	36653-82-4	2308	1.108	1887	1882	83	822
1-Heptadecanol	1454-85-9	2536	1.088	1976	1986	111	864
Aromatic Compounds							
Benzene	71-43-2	260	0.788	656	658	78	954
Toluene	108-88-3	436	1.012	769	766	91	836
Ethylbenzene	100-41-4	644	0.960	864	857	91	948
1,2-Dimethylbenzene (o-Xylene)	95-47-6	664	0.960	872	886	91	949
Styrene (Ethenylbenzene)	100-42-5	712	1.260	893	891	104	945
2-Propenylbenzene (Allyl benzene)	300-57-2	836	1.060	950	953	118	838
1-Ethyl-2-methylbenzene (2-Ethyltoluene)	611-14-3	864	0.928	963	973	105	822
1-Ethyl-4-methylbenzene (4-Ethyltoluene)	622-96-8	892	0.940	976	969	105	907
(1-Methylethenyl)benzene (Isopropenylbenzene)	98-83-9	912	1.132	985	988	118	870
Methoxymethylbenzene (alpha-Methoxytoluene)	538-86-3	924	1.320	991	984	91	926
1,2,3-Trimethylbenzene (Hemimellitene)	526-73-8	936	0.976	996	1018	120	835
1-Methyl-3-vinylbenzene (m-Methylstyrene)	100-80-1	940	1.164	998	973	118	916
1-Ethenyl-2-methylbenzene (2-Methylstyrene)	611-15-4	948	1.152	1002	991	118	903
Cyclopropylbenzene (Phenylcyclopropane)	873-49-4	972	1.128	1014	1010	118	905
1-Methoxy-4-methylbenzene (4-Methoxytoluene, 4-Methylanisole, p-Methylanisole)	104-93-8	992	1.328	1024	1022	122	914
1-Methyl-4-propylbenzene (4-Propyltoluene)	1074-55-1	1064	0.888	1061	1056	105	811
Butylbenzene (1-Phenylbutane)	104-51-8	1068	0.896	1063	1068	92	800
1-Ethyl-2,4-dimethylbenzene (4-Ethyl-m-xylene)	874-41-9	1116	0.944	1088	1084	119	785
1-Vinyl-3-ethylbenzene (3-Ethylstyrene)	7525-62-4	1120	1.080	1090	1064	117	944
1-Ethenyl-4-ethylbenzene (4-Ethylstyrene, p-Ethylstyrene)	3454-07-7	1140	1.076	1100	1072	117	936
1,3-Diethenylbenzene (m-Vinylstyrene)	108-57-6	1176	1.344	1120	1091	130	923
1-Methoxy-4-vinylbenzene (4-Methoxystyrene, 4-Vinylanisole)	637-69-4	1248	1.588	1159	1159	134	887
Naphthalene	91-20-3	1312	1.688	1193	1179	128	833
1-Methoxy-4-(2-propenyl)benzene (4-Allylanisole, Estragole)	140-67-0	1328	1.356	1202	1201	148	945
1-Methoxy-4-propenylbenzene (Anethole, Isoestragole)	104-46-1	1424	1.404	1260	1288	148	951
Hexylbenzene	1077-16-3	1436	0.868	1267	1251	92	820

1-Methoxy-4-propenylbenzene (Anethole, Isoestragole)	104-46-1	1480	1.512	1293	1288	148	949
1-Methylnaphthalene	90-12-0	1504	1.560	1308	1296	141	812
1,1'-Biphenyl (Lemonene, Phenylbenzene)	92-52-4	1636	1.672	1390	1380	154	926
1,2-Dimethoxy-4-(2-propenyl)benzene (1,2-Dimethoxy-4-allyl benzene, Methyl eugenol)	93-15-2	1656	1.660	1403	1403	178	922
2-Methyl-1,1'-biphenyl (2-Phenyltoluene)	643-58-3	1660	1.380	1405	1395	167	902
1,4-Dimethylnaphthalene	571-58-4	1696	1.552	1429	1423	141	792
1,3-Dimethylnaphthalene	575-41-7	1704	1.528	1434	1425	141	860
4-Methyl-1,1'-biphenyl	644-08-6	1792	1.580	1492	1493	168	923
1,2-Dimethoxy-4-[(1E)-1-propenyl]benzene (trans-4-Propenylveratrole, trans-Methylisoeugenol)	6379-72-2	1800	1.836	1497	1495	178	880
4-Methyl-1,1'-biphenyl	644-08-6	1808	1.580	1503	1493	168	918
(2-Phenylethyl)benzene (Dihydrostilbene)	103-29-7	1844	1.476	1529	1520	91	939
(1-Butylheptyl)benzene	4537-15-9	1992	0.812	1636	1626	91	763
(1-Pentylheptyl)benzene (6-Phenyldodecane)	2719-62-2	2116	0.812	1731	1719	91	834
(1-Propylnonyl)benzene (4-Phenyldodecane)	2719-64-4	2140	0.820	1750	1735	91	782
Saturated Carboxylic Acids							
Acetic acid	64-19-7	348	1.112	723	709	45	893
Nonanoic acid	112-05-0	1444	1.132	1271	1273	87	812
Dodecanoic acid	143-07-7	1888	3.472	1560	1558	73	881
Tetradecanoic acid	544-63-8	2152	2.856	1759	1760	60	894
Pentadecanoic acid	1002-84-2	2276	2.656	1860	1851	60	836
Hexadecanoic acid	57-10-3	2396	2.452	1926	1925	60	915
Heptadecanoic acid	506-12-7	2616	2.268	2007	1977	60	na
Aromatic Aldehydes							
Benzaldehyde (Phenylmethanal)	100-52-7	872	2.284	967	964	106	939
Benzeneacetaldehyde (2-Phenylethanal, Phenylacetaldehyde)	122-78-1	1040	2.280	1049	1045	91	886
4-Methylbenzaldehyde (p-Methoxybenzaldehyde, p-Tolualdehyde)	104-87-0	1092	1.896	1076	1076	91	849
2-Phenylpropenal (Atropaldehyde)	4432-63-7	1252	2.284	1161	1161	103	787
4-Ethylbenzaldehyde (p-Ethylbenzaldehyde)	4748-78-	1268	1.684	1170	1164	134	889

	1						
4-Ethylbenzaldehyde (p-Ethylbenzaldehyde)	4748-78-1	1300	1.720	1187	1164	134	915
4-Methoxybenzaldehyde (4-Anisaldehyde)	123-11-5	1428	2.924	1262	1252	135	936
(2E)-2-Methyl-3-phenyl-2-propenal (alpha-Methylcinnamaldehyde)	101-39-3	1456	1.876	1279	1309	146	833
Aromatic Ketones							
1-(4-Methylphenyl)ethanone (1-Methyl-4-acetylbenzene, 4'-Methylacetophenone)	122-00-9	1308	1.868	1191	1183	119	819
1-(4-Ethylphenyl)ethanone (4'-Ethylacetophenone, p-Acetylethylbenzene)	937-30-4	1476	1.704	1290	1274	133	874
1,4-Diacetylbenzene (p-Acetylacetophenone)	1009-61-6	1708	3.340	1437	1451	91	870
1-(4-Methoxyphenyl)-1-propanone (4'-Methoxypropiophenone, p-Methoxypropiophenone)	121-97-1	1736	2.268	1455	1484	135	756
1,4-Diacetylbenzene (p-Acetylacetophenone)	1009-61-6	1736	3.404	1455	1451	147	880
Benzophenone (Diphenylmethanone)	119-61-9	1996	2.372	1639	1635	105	891
Benzyl Alcohols							
Benzenemethanol (Benzyl alcohol, Phenylmethanol)	100-51-6	1032	2.064	1045	1037	108	838
Benzeneethanol (2-Phenylethanol, Mellol)	60-12-8	1180	0.168	1122	1118	92	936
4-Methoxybenzenemethanol (Anisyl alcohol)	105-13-5	1480	1.832	1293	1295	138	755
Alkanes							
Tridecane	629-50-5	1500	0.648	1305	1300	57	926
Tetradecane	629-59-4	1660	0.660	1405	1400	57	933
Pentadecane	629-62-9	1808	0.664	1503	1500	57	911
Hexadecane	544-76-3	1948	0.672	1603	1600	57	897
Heptadecane	629-78-7	2084	0.672	1706	1700	57	897
Benzyl Acetates							
Benzyl acetate (Phenylmethyl acetate, (Acetoxymethyl)benzene)	140-11-4	1264	1.784	1167	1167	108	950
Phenylethyl acetate	103-45-7	1424	1.632	1260	1257	104	904
Glycol Ethers							
2-Ethoxyethanol	110-80-5	444	2.188	773	744	59	889
2-Butoxyethanol	111-76-2	748	2.012	909	903	57	871
2-(2-Ethoxyethoxy)ethanol (Diethylene glycol monoethyl ether)	111-90-0	968	2.580	1012	1006	45	940

$^{1}t_R$ – first dimension retention time
$^{2}t_R$ – second dimension retention time
RI_{exp} – experimental linear temperature-programmed retention index
RI_{lit} – literature linear temperature-programmed retention index
SIM – mass spectral similarity

Table 3. Metabolites unique to *in vivo* DI-SPME sampling approach

analyte name (hit # 1)	CAS # (hit # 1)	$^{1}t_R$; sec	$^{2}t_R$; sec	RI_{exp}	RI_{lit}	unique mass	SIM (hit # 1)
**unidentified analyte 1 (hit # 1: 1-Hepten-4-ol)**	3521-91-3	1752	3.540	1466	na	55	762
**unidentified analyte 2 (hit # 1: 2-(4-tert-Pentylphenoxy)ethanol)**	6382-07-6	2280	2.176	1863	na	179	769
**1,4-Diacetylbenzene (p-Acetylacetophenone)**	1009-61-6	1736	3.404	1455	1451	147	880

$^{1}t_R$ – first dimension retention time
$^{2}t_R$ – second dimension retention time
RI_{exp} – experimental linear temperature-programmed retention index
RI_{lit} – literature linear temperature-programmed retention index
na – not available
SIM – mass spectral similarity
